# Tailoring Supramolecular Polyurethane Featuring Bio-based Rigid–Flexible Segment Hybrids with Intrinsic Photothermal Conversion and Ultraviolet Blocking

**DOI:** 10.34133/research.1282

**Published:** 2026-05-12

**Authors:** Yun Hu, Ye Sha, Yan Fang, Xingxiang Liu, Meng Zhang, Puyou Jia, Guodong Feng, Liang Yuan, Lin Dai, Yonghong Zhou

**Affiliations:** ^1^ Institute of Chemical Industry of Forest Products, Chinese Academy of Forestry, Nanjing 210042, China.; ^2^ Department of Chemistry and Materials Science, College of Science, Nanjing Forestry University, Nanjing 210037, China.; ^3^ College of Chemical Engineering, Nanjing Forestry University, Nanjing 210037, China; ^4^ Anhui Provincial Engineering Center for High Performance Biobased Nylons, Biomass Molecular Engineering Center, School of Materials and Chemistry, Anhui Agricultural University, Hefei, Anhui 230036, China.; ^5^ State Key Laboratory of Bio-based Fiber Materials, Tianjin Key Laboratory of Pulp and Paper, College of Light Industry and Engineering, Tianjin University of Science and Technology, Tianjin 300457, China.

## Abstract

Multifunctional polyurethanes face a fundamental design trade-off: enhanced cross-linking and rigidity improve mechanical robustness but inevitably restrict chain mobility, thereby compromising essential features such as self-healing and recyclability. This inherent compromise severely constrains their multifunctional application. Here, we report a supramolecular polyurethane (COPUSL) comprising a “dynamic switch” constructed from dynamic disulfide bonds and hydrogen bonds, together with a “rigid–flexible balanced network” composed of castor oil long fatty chains and polyphenol-functionalized lignin. This design endows COPUSL with excellent mechanical properties while also enabling rapid self-healing (self-healing efficiency: 87%) and efficient recyclability. Furthermore, COPUSL with introduced aromatic structures and extended conjugate systems exhibits 100% ultraviolet-blocking efficiency and a high photothermal conversion capability (surface temperature: 153 °C) due to the electron transition and energy release of the lignin structure after absorbing light energy. By systematically investigating the relaxation kinetics, dynamic behavior, and macroscopic properties, we elucidate the distinct roles of the “dynamic switch” and “rigid–flexible balanced network” in regulating the polymer architecture and connecting dynamic behavior with mechanical and functional performance. These findings provide molecular-level insights for the design of high-performance, bio-based polyurethane with tailored multifunctional responsiveness.

## Introduction

Polyurethanes, with their unique chain architecture and excellent overall mechanical properties, show great potential in emerging fields such as aerospace, soft robotics, flexible electronics, and stretchable optical devices [1–8]. The growing demand for sustainable and intelligent materials has facilitated the development of self-healing and recyclable polyurethanes from renewable raw materials [9–13]. By partially or fully replacing petroleum-based feedstocks with biomass resources, bio-based polyurethanes have become a research focus and achieved remarkable progress [14–22]. It has been reported that castor oil, palm oil, and natural rubber were used as polyols to prepare bio-based polyurethanes [23–25]. Some bio-based polyurethanes can surpass the performance of conventional petroleum-based counterparts, providing an important pathway toward green, low-carbon, and sustainable development [26,27]. However, the development of polyurethane that integrates adjustable mechanical strength, rapid self-healing, recyclability, and multifunctional performance remains fundamentally limited by a critical trade-off, because enhancing mechanical properties through increased cross-linking and rigid structures typically impedes the chain mobility essential for self-healing and recycling, which further constrains the application of polyurethanes in photothermal conversion and ultraviolet (UV) blocking.

Polyurethane photothermal conversion materials have drawn considerable interest for intelligent applications [28]. Typically, photothermal agents such as carbon-based materials and semiconducting metal oxides are incorporated into polyurethane matrices to impart photothermal responsiveness [29,30]. However, achieving uniform and stable dispersion of these photothermal agents within the polymer remains challenging, and many efficient photothermal additives are costly [31,32]. As the most abundant natural aromatic polymer, lignin offers a promising alternative due to its polyhydroxyl groups, phenyl rings, and capacity for π–π stacking [33,34], which also enable it to function as a natural photothermal agent [35,36]. The π–π conjugated network within lignin’s aromatic units enables efficient light absorption and photothermal conversion [37,38]. For example, through in situ via zinc-ion coordination and hydrogen bonding, a fully bio-based elastomer achieved a photothermal conversion efficiency of 69.47% [39]. Our team have developed a lignin–tung-oil-based covalent adaptable network photothermal material that exhibits strong mechanical properties, stable photothermal response, and good recyclability [40]. Nevertheless, reports on polyurethane that combine rigid–flexible hybrid segment architectures with dynamic cross-linked networks, especially for multifunctional application in UV blocking and photothermal conversion, remain scarce.

**Fig. 1.**
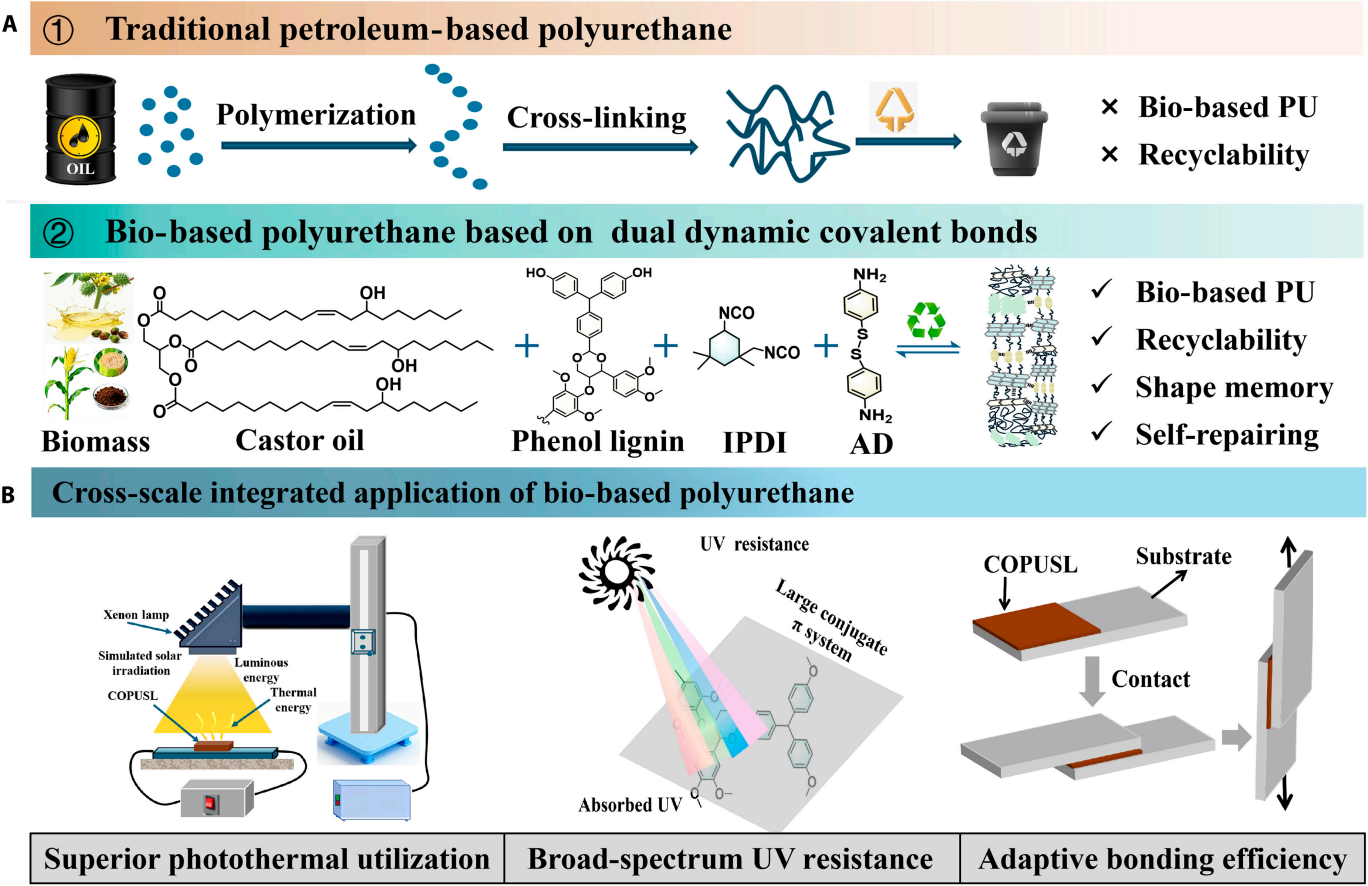
(A) Traditional petroleum-based polyurethane and supramolecular polyurethane comprising a “dynamic switch” and a “rigid–flexible balanced network”. (B) Cross-scale integrated application of the bio-based polyurethane.

Here, we designed a supramolecular polyurethane (COPUSL) with intrinsic photothermal conversion and UV blocking, which comprises a “dynamic switch” constructed from dynamic disulfide bonds and hydrogen bonds, together with a “rigid–flexible balanced network” composed of castor oil long fatty chains and polyphenol-functionalized lignin. This structure effectively balances the mechanical performance, recyclability, and multifunctional characteristics of COPUSL. Specifically, COPUSL was synthesized from castor oil and phenol lignin derived from corncob residue after xylose extraction (Fig. 1). We conducted a comprehensive investigation into the molecular chain dynamics, self-healing ability, recyclability, and shape-memory behavior of the resulting polyurethane. Furthermore, by integrating molecular dynamics simulations and density functional theory (DFT) calculations, we substantiated the role of the “dynamic switch” and “rigid–flexible balanced network” in modulating the rigid–flexible interactions within the polyurethane chain segments. Leveraging the intrinsic properties of lignin, COPUSL was directly developed as a UV-shielding and integrated photothermal–electric converter, with its anti-UV performance and photothermal conversion efficiency systematically evaluated.

## Results and Discussion

To balance the regulated mechanical performance, recyclability, and multifunctional features of polyurethane, we designed a supramolecular polyurethane with intrinsic photothermal conversion and UV blocking based on a “dynamic switch” and “rigid–flexible balanced network” architecture. The “dynamic switch” is constructed from dynamic disulfide bonds and hydrogen bonds to endow materials with self-healing, reshaping, and recyclability, while the “rigid–flexible balanced network” is composed of castor oil long fatty chains and polyphenol-functionalized lignin to control mechanical properties [10]. This architecture, incorporating a dynamic switch” and a “rigid–flexible balanced network”, offers a promising approach to addressing the trade-off between mechanical strength and self-healing ability. Specifically, using corncob residue from industrial xylose extraction as the raw material, functionalized lignin was prepared via the *p*-phthalaldehyde protection method (TALD). This process involved the reaction of aldehyde groups with phenols under acidic conditions, which facilitated the further phenolation of TALD lignin. This treatment increased the phenolic hydroxyl content [41]. ^1^H nuclear magnetic resonance (NMR), Fourier transform infrared spectroscopy (FT-IR), heteronuclear single quantum coherence NMR, and ^31^P NMR confirmed the successful modification (Figs. S1 to S4).

**Fig. 2.**
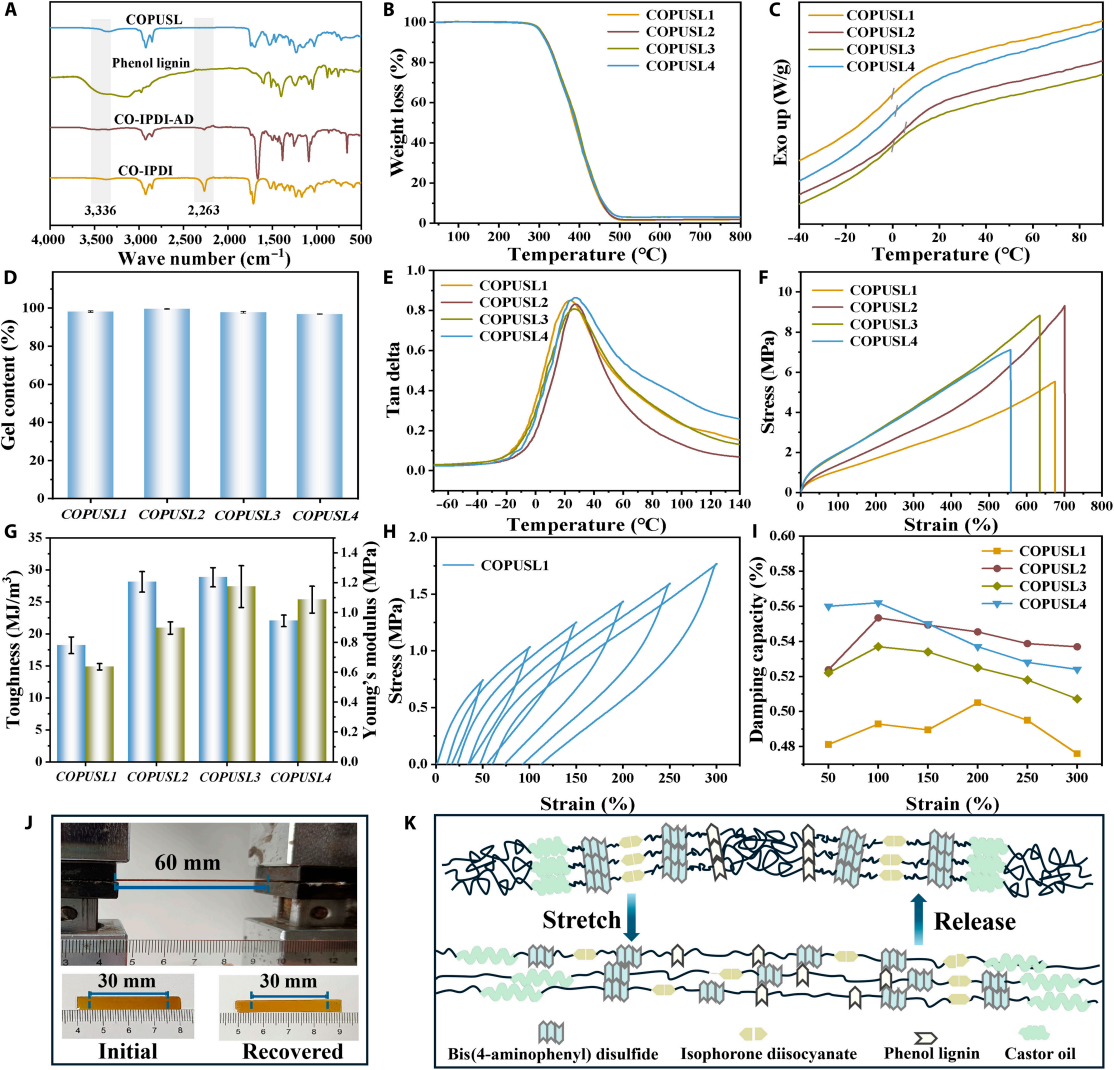
(A) Fourier transform infrared spectroscopy (FT-IR) spectra of COPUSL and the intermediates. (B) Thermogravimetric analysis (TGA) curves of COPUSL. (C) Differential scanning calorimetry (DSC) curves of COPUSL. (D) Gel content of COPUSL. (E) Loss factor curves of COPUSL. (F) Tensile stress–strain curves of COPUSL. (G) Toughness (blue) and Young’s modulus (tawny) of COPUSL. (H) Cyclic tensile curves of COPUSL1. (I) Damping efficiency via cyclic tensile tests of COPUSL. (J) Resilience test of COPUSL1. (K) Schematic elastic recovery mechanism of COPUSL.

The synthesized phenol lignin was then used to prepare polyurethane prepolymers (Fig. 2A). Following the addition of bis(4-aminophenyl) disulfide (AD) to the castor oil precursor (COIPDI), a characteristic absorption peak emerged at 1,594 cm^−1^ (N–H vibration). Upon subsequent incorporation of phenol lignin, the characteristic absorption peak of the carbonyl group originally located at 1,717 cm^−1^ shifts to 1,698 cm^−1^ due to a redshift caused by hydrogen bonding (Fig. S5). The broad peak at 3,417 cm^−1^ corresponds to the hydroxyl groups of phenol lignin, confirming their retention in the polymer structure. Meanwhile, the absorption at 3,336 cm^−1^ is assigned to the –NH– stretching of the carbamate linkage, verifying successful urethane formation. In the FT-IR spectrum of COIPDI, the –NCO stretching band at 2,263 cm^−1^ was weaker than that of isophorone diisocyanate [42]. With the sequential addition of AD and phenol lignin, this peak finally vanished, demonstrating complete consumption of the –NCO groups. Collectively, the FT-IR analysis provided robust evidence for the successful synthesis of the target polyurethane prepolymers.

The thermal stability of COPUSL showed that the initial degradation temperatures (*T*_i_) of COPUSL*X* (where *X* = 1, 2, 3, and 4) were all above 303 °C (Fig. 2B and Fig. S6), confirming excellent thermal stability. The glass transition temperature (*T*_g_) of COPUSL ranged from 0.04 to 5.37 °C; the high *T*_g_ is caused by the high cross-linking density (Fig. 2C). The x-ray diffraction patterns of COPUSL displayed 2 broad diffraction peaks at 2*θ* = 19° and 41° (Fig. S7). This amorphous characteristic is more conducive for the formation of transparent films, rendering COPUSL suitable for photothermal conversion applications. The gel content (*C*_gel_) of COPUSL2 achieved the highest value of 99.47%, indicating a high cross-linking degree, but excess and unevenly dispersed polyphenol lignin caused a low cross-linking degree (Fig. 2D and Table S2). The cross-linking density (*υ*_e_) of COPUSL was also evaluated (Fig. 2E and Fig. S8). At 25 °C, the storage modulus (*E*_25_) of COPUSL peaked at 81.3 MPa for COPUSL3. *υ*_e_ is calculated using the following equation:

$$\upsilon_{e}=E/\left(3\mathrm{RT}\right)$$ (1)

where *E* denotes the storage modulus at *T*_g_ + 50 °C, *R* is the universal gas constant, and *T* is the absolute temperature. Calculations revealed that COPUSL3 exhibited the highest *υ*_e_ (1,220.41 mol/m^3^). *υ*_e_ showed an initial increase followed by a decrease, which was consistent with the variation of *C*_gel_, indicating that the addition of polyphenol lignin can increase the *υ*_e_ of COPUSL, but excess and unevenly dispersed polyphenol lignin inhibits thermosetting cross-linking. As the lignin content increased, the surface microstructure gradually transitioned from smooth to rough, indicating that excessive lignin compromises the homogeneity (Fig. S9).

With increasing phenol lignin content, the tensile strength and elongation at break of COPUSL firstly increased and then decreased, which is consistent with the changes in *υ*_e_ (Fig. 2F and G and Fig. S10). The mechanical behavior of COPUSL can be regulated due to the “rigid–flexible balanced network” [43]. A comparative analysis with analogous systems was conducted (Fig. S11), which highlights the superior mechanical properties of the presented material. To evaluate the elastic recovery capability, continuous cyclic tensile tests were performed on COPUSL within a maximum strain range of 50% to 300% (Fig. 2H, Table S3, and Fig. S12). Distinct hysteresis loops were observed for COPUSL, and increasing tensile strain resulted in the expansion of these loops, indicating that strain energy can be efficiently dissipated via the “dynamic switch”. COPUSL exhibited increasing damping capacities (Fig. 2I). This trend indicated that the rupture of hydrogen bonds in COPUSL4 during stretching dissipates more energy, thereby enhancing toughness. Even after being stretched to twice its original length, COPUSL2 fully recovered to its initial dimensions after a 1-min resting period (Fig. 2J), confirming that COPUSL possessed excellent elastic recovery capability.

COPUSL exhibited exceptional self-healing properties, attributed to the reformation of hydrogen bond networks (Fig. 2K). Rheological tests provided insights into the structural evolution and viscoelastic behavior of COPUSL (Fig. S13). Within a frequency range of 0.001 to 1 Hz, the storage modulus (*G*′) of COPUSL remained consistently higher than the loss modulus (*G*′′) across different temperatures, indicating a thermoset state and that the cross-linked network remained intact under shear stress [44]. The temperature variations primarily affect dynamic exchange kinetics without altering the samples’ cross-linking density [45]. Additionally, rheological measurements revealed that COPUSL did not undergo phase transitions at 120 °C and retained a solid state (Fig. S14), a property favorable for subsequent reprocessing and self-healing.

**Fig. 3.**
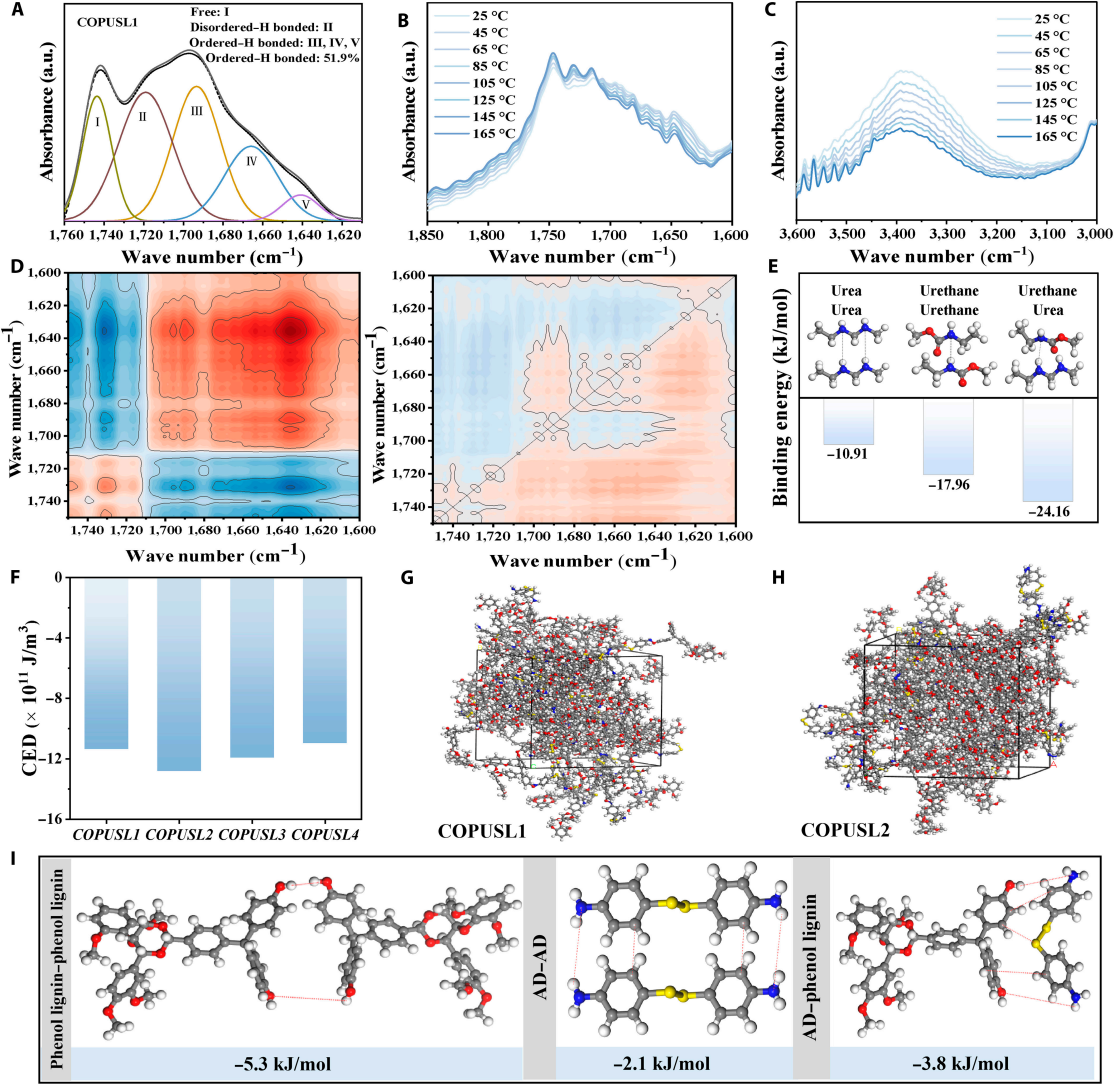
(A) Fourier transform infrared spectroscopy (FT-IR) spectra of the C=O stretching vibration region in COPUSL1. (B and C) Variable-temperature FT-IR spectra of COPUSL. (D) 2-dimensional FT-IR results of COPUSL1: the synchronous spectrum and the asynchronous spectrum. (E) Calculated binding energies of various hydrogen bonds in COPUSL. (F) Cohesive energy density (CED) of COPUSL calculated via molecular dynamics (MD) simulations. (G and H) Kinetic simulation visualized models of COPUSL1 and COPUSL2. (I) Density functional theory (DFT)-assisted binding energy of the bis(4-aminophenyl) disulfide (AD)–phenol lignin dimer, phenol lignin–phenol lignin dimer, and AD–AD dimer.

The C=O stretching vibration region of COPUSL1 was deconvoluted into 5 peak values through peak fitting (Fig. 3A), corresponding to the free state (1,743 cm^−1^), disordered state (1,719 cm^−1^), and ordered hydrogen-bonded states (1,693, 1,665, and 1,640 cm^−1^) [46]. Similar peak fitting was performed for COPUSL2 to COPUSL4 (Fig. S15 and Table S4). COPUSL4 exhibited the highest hydrogen bonding ratio (68.9%), with a greater proportion of ordered hydrogen bonds contributing to stronger hydrogen bonding interactions [39]. Variable-temperature FT-IR analysis was carried out for a deeper understanding of the hydrogen bonds (Fig. 3B and C). A broad –NH stretching vibration absorption band was observed in the 3,100- to 3,500-cm^−1^ range, attributed to carbamate and urea groups [47]. As the temperature increased, the intensity of the free –NH peak was enhanced, while that of the hydrogen-bonded –NH peak was diminished. Concurrently, the characteristic free C=O peak displayed an increased intensity and a blueshift. These changes provide direct evidence for the thermally induced dissociation of hydrogen bonds [48]. The same phenomena were observed for COPUSL2 to COPUSL4 (Fig. S16), clearly demonstrating the presence of hierarchical hydrogen bonding in COPUSL at the molecular scale, an interaction that plays a critical role in energy dissipation during stretching. In the 2-dimensional correlation maps (Fig. 3D and Fig. S17), pink and blue regions represent positive and negative changes in spectral band intensity, respectively [49]. According to Noda’s rule [50], the peaks at 1,730, 1,700, and 1,630 cm^−1^ were found to shift progressively from 1,730, 1,700, and 1,630 cm^−1^, confirming the presence of hierarchical hydrogen bonding interactions in COPUSL.

DFT calculations were employed to further quantify the binding energies of the hydrogen bonds in COPUSL (Fig. 3E). The strengths of these hydrogen bonds follow the order carbamate–urea hydrogen bonds (−24.16 kJ·mol^−1^) > urea–urea hydrogen bonds (−17.96 kJ·mol^−1^) > carbamate–carbamate hydrogen bonds (−10.91 kJ·mol^−1^). The relatively weaker hydrogen bonds act as dynamic weak cross-links, preferentially dissociating to dissipate energy during stretching, thereby endowing the material with excellent tensile ductility [51]. In contrast, the stronger hydrogen bonds constrain the relaxation of the stretched “rigid–flexible balanced network” under large strains, markedly enhancing the material’s stretchability and toughness. Owing to the meticulously designed hierarchical hydrogen bonding network, COPUSL exhibits outstanding comprehensive mechanical properties and achieves simultaneous reinforcement of strength and toughness.

The cohesive energy density of systems with different AD and phenol lignin molar ratios was obtained from molecular dynamics simulations. To simplify the calculations, only AD and phenol lignin chains were used as modeling units (Fig. 3F to H and Fig. S18) [52]. COPUSL2 exhibited the highest cohesive energy density (Fig. 3F). DFT calculations of dimer binding energies confirmed that the lignin–phenol dimer has the highest binding energy (Δ*E* = −5.3 kJ·mol^−1^), the AD–AD dimer has the lowest binding energy (Δ*E* = −2.1 kJ·mol^−1^), and the AD phenol lignin dimer binding energy (Δ*E* = −3.8 kJ·mol^−1^) falls between the 2 (Fig. 3I), indicating that an excess of rigid phenol lignin structures leads to excessively strong intermolecular interactions, while an overabundance of AD structures significantly weakens the noncovalent interactions [53]. The results demonstrated that the “rigid–flexible balanced network” based on castor oil long fatty chains and polyphenol-functionalized lignin can effectively modulate intermolecular interactions, thereby regulating the mechanical performance of COPUSL. Furthermore, the influence of hydrogen bonding on mechanical properties was quantitatively assessed through peak deconvolution according to our previous studies [54,55].

**Fig. 4.**
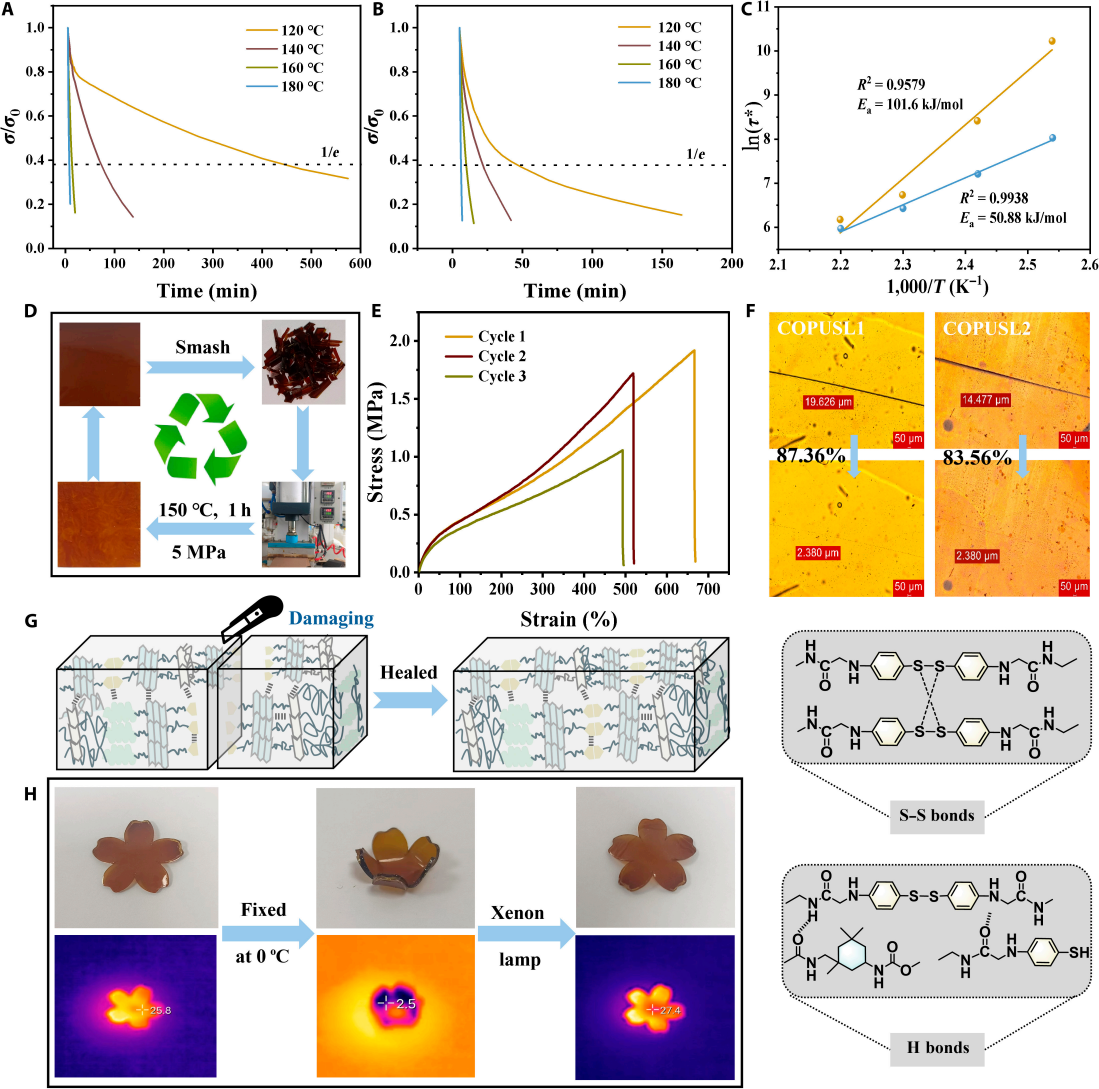
(A and B) Stress relaxation curves of COPUSL2 and COPUSL3. (C) Activation energy of COPUSL2 and COPUSL3 derived from the Arrhenius equation. (D) Schematic diagram of hot-press recycling. (E) Stress–strain curves of COPUSL2 after multiple recycling processes. (F) Images of COPUSL1 and COPUSL2 before and after self-healing under heating at 120 °C for 10 min. (G) Schematic diagram of the self-healing mechanism of COPUSL. (H) Shape-memory images of COPUSL3 and images recorded via infrared thermal imaging during xenon lamp irradiation.

Stress relaxation tests were performed to validate the kinetics of dynamic bond exchange and topological rearrangement (Fig. 4A and B). For COPUSL2, the characteristic relaxation times (τ^*^) were 468.3 min at 120 °C and 7.5 min at 180 °C, respectively. As the temperature increased, the dynamic exchange rate of S–S bonds accelerated, and elevated temperatures reduced the time required for topological chain rearrangement. The τ^*^ values of COPUSL closely follow the Arrhenius relationship, which was used to calculate the activation energy (*E*_a_) [56]:

$$\ln\;\tau^{*}=E_{a}\;/\;\left(\mathrm{RT}\right)\;-\;\ln\;A$$ (2)

where *T* is the absolute temperature, *A* denotes the frequency factor, and *R* is the molar gas constant. The *E*_a_ values for COPUSL2 and COPUSL3 were determined to be 101.6 and 50.88 kJ/mol, respectively (Fig. 4C). This confirms the formation of a dual-cross-linked network in COPUSL, consisting of a “dynamic switch” based on hydrogen bonds and dynamic disulfide bonds.

COPUSL2 and COPUSL3 were crushed into fragments to investigate reprocessability (Fig. 4D); the fragments were hot-pressed and formed rectangular specimens for tensile testing (Fig. 4E and Fig. S19). The reduction of mechanical properties was attributed to high-temperature side reactions including hydrolysis or oxidation of carbamate and urea bonds. Considering time and cost, 10 min is the optimal self-healing time (Fig. S20). The self-healing capability of COPUSL reached 87% after healing due to the transformation of the “dynamic switch” (Fig. 4F and G and Fig. S21) [57]. In lignin-derived polyurethane elastomers, the lignin structure usually serves both as rigid cross-linking points and as a reinforcing filler, thereby inducing a strain-hardening effect. The incorporation of lignin influences the stress relaxation process, which in turn affects the self-healing efficiency [35]. COPUSL3 was cut into a petal shape to investigate the shape-memory behavior. When the fixed COPUSL3 was exposed to xenon lamp irradiation, the petals gradually recovered their original shape as the temperature increased (Fig. 4H and Fig. S22). The quantitatively calculated shape fixation rate of COPUSL1 (*R*_f_) was 100%, and the shape recovery rate (*R*_r_) was 74% (Figs. S23 to S26 and Table S5). The solvent resistance of COPUSL was assessed against various solvents including toluene, water, ethyl acetate, ethanol, petroleum ether, sulfuric acid, 1 mol/l NaOH, and 1 mol/l HCl (Fig. S27). After 7 d of immersion, no significant change was observed in water, petroleum ether, or acidic/alkaline solutions, indicating excellent resistance.

**Fig. 5.**
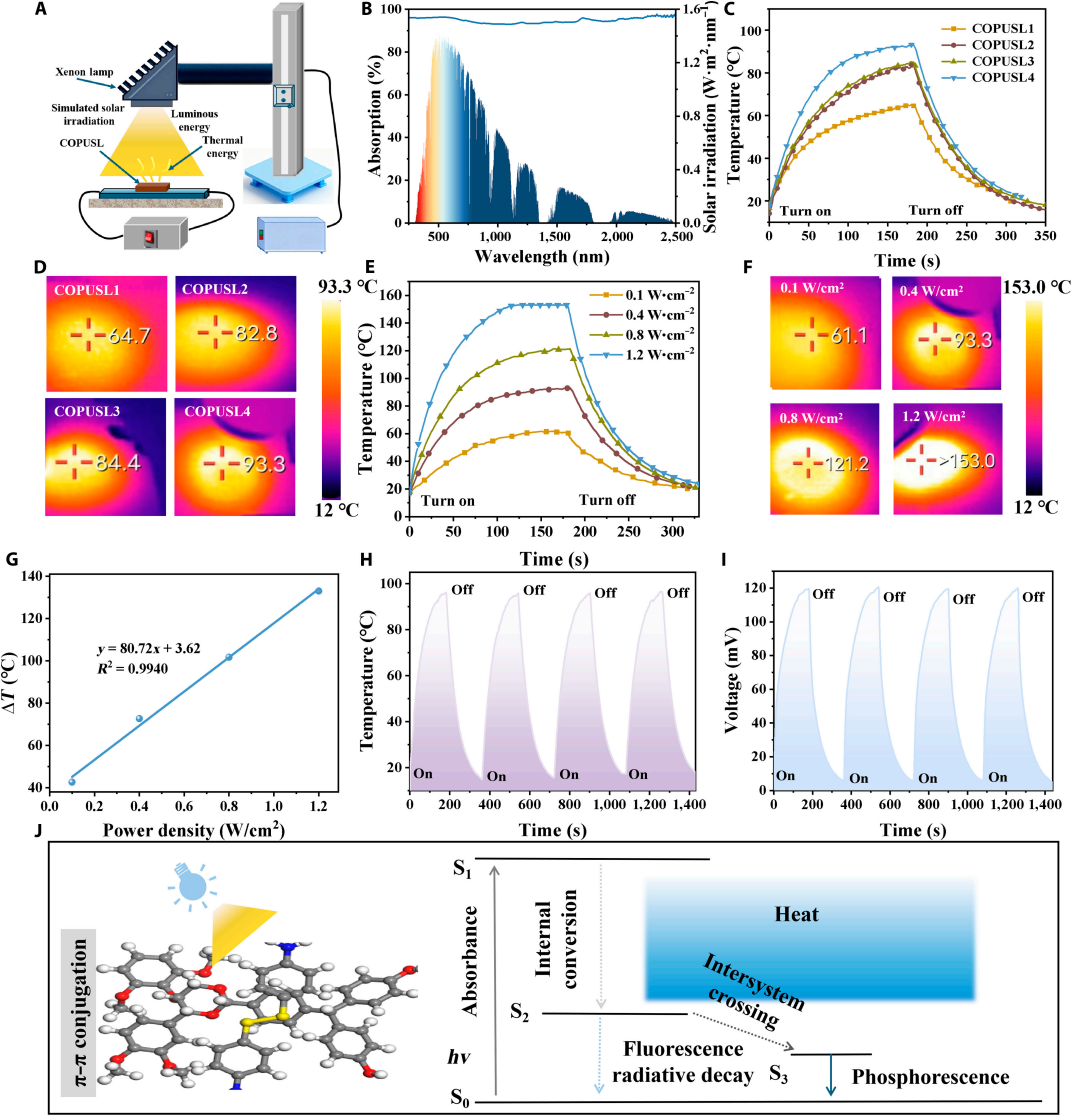
(A) Schematic diagram of the photothermal test for COPUSL. (B) Ultraviolet–visible–near-infrared (UV–Vis–NIR) diffuse reflectance spectra of COPUSL4. (C and D) Temperature changes and infrared thermal images of COPUSL under 0.4 W/cm^2^ xenon lamp irradiation for 180 s. (E and F) Temperature changes and infrared thermal images of COPUSL4 under 0.1, 0.2, 0.3, and 0.4 W/cm^2^ xenon lamp irradiation. (G) Curve of maximum temperature increase (Δ*T*) versus light intensity of COPUSL4. (H and I) Temperature changes and open-circuit voltage variations of COPUSL4 during 4 on–off cycles under 1.2 W/cm^2^ xenon lamp irradiation. (J) Photothermal conversion mechanism of COPUSL.

COPUSL inherits outstanding photothermal performance, enabled by the π–π stacking ability of lignin molecules, which affords efficient photon harvesting and rapid thermal conduction. The surface temperature evolution of COPUSL under xenon lamp irradiation was recorded using an infrared thermal camera, enabled by the π–π stacking ability of lignin molecules (Fig. 5A).

UV–visible–near-infrared spectra confirmed that COPUSL4 achieved ultrahigh light absorption (up to 95%) across the entire solar spectrum (Fig. 5B). COPUSL was irradiated under a xenon lamp at an intensity of 400 mW/cm^2^, with temperature changes recorded via infrared thermography (Fig. 5C). Within 3 min of irradiation, the surface temperature of COPUSL rose to 93.3 °C (Fig. 5D). When COPUSL4 was exposed to light intensities of 100, 400, 800, and 1,200 mW/cm^2^, the maximum temperature (*T*_max_) exceeded 153 °C; the photothermal conversion performance is comparable to that of a reported lignin-based waterborne polyurethane elastomer (Fig. 5E and F) [58]. The temperature elevation (Δ*T*) exhibited a linear correlation with increasing light intensity (Fig. 5G). These results demonstrated that the *T*_max_ of the films can be precisely regulated by adjusting the phenol lignin content and light intensity. The stability and multifunctionality of COPUSL were evaluated by 4 on–off cycle tests under a light intensity of 1,200 mW/cm^2^ (Fig. 5H), which remained stable across multiple cycles. A universal electric meter was used to measure the open-circuit voltage during photothermal conversion, which reached 120 mV. The voltage signal was found to synchronize with temperature fluctuations (Fig. 5I), confirming the excellent stability of COPUSL in photothermal–electrical conversion.

The photothermal conversion mechanism is primarily attributed to the naturally occurring conjugated carbonyl groups, double bonds, and quinone structures within its benzene rings [38]. The π–π conjugated system of phenol lignin narrows the energy gap: as COPUSL absorbs light, loosely bound electrons in its structure readily undergo π–π* transitions. When the energy of incident photons matches the electronic transition requirements of phenol lignin, the energy barrier for electrons to shift from the ground state (S_0_) to the excited state (S_1_) is effectively reduced (Fig. 5J). Subsequent nonradiative relaxation of electrons back to the ground state releases energy in the form of heat, thereby converting light energy into thermal energy. The high-density hydrogen bonds of COPUSL molecules (calculated by DFT in Fig. 3E) formed a dense polymer network. This densification enhances intermolecular stacking interactions, thereby increasing π–π conjugation between benzene rings. The reinforcement lowers the energy barrier for electronic transitions and improves the overall light absorption and conversion efficiency of COPUSL. Compared with typical additive-based types, the intrinsic polymer in this study exhibits superior photothermal conversion efficiency (Fig. S28).

**Fig. 6.**
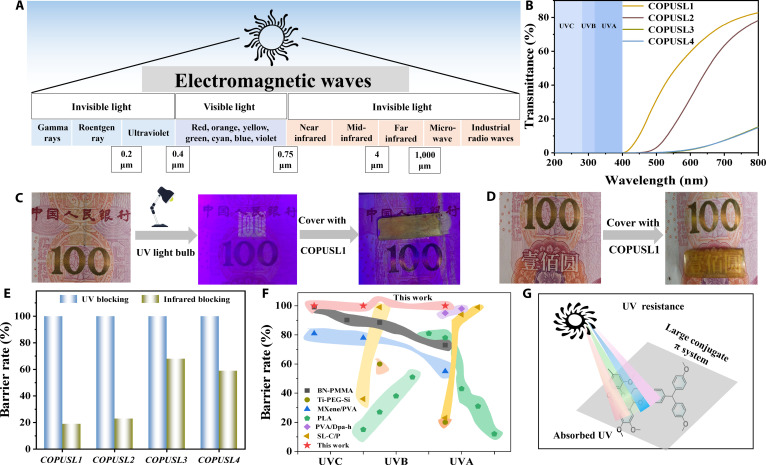
(A) Solar emission spectral wavelengths. (B) Transmittance of COPUSL in the 200- to 800-nm range. (C) Demonstration diagram of the ultraviolet (UV)-blocking process for COPUSL. (D) Visual appearance of COPUSL under visible light. (E) UV and infrared blocking rates of COPUSL. (F) Comparison of blocking rates between COPUSL and the literature. (G) UV resistance mechanism of COPUSL.

COPUSL exhibits effective full-spectrum UV protection (UVA, UVB, and UVC) due to the presence of aromatic rings and oxygen atoms with lone-pair electrons in the “rigid–flexible balanced network” based on the lignin structure. COPUSL1 and COPUSL2 demonstrated a visible light transmittance of approximately 80%, which indicates the amorphous structure of COPUSL1 (Fig. 6A and B) [59], which is consistent with the DSC results. COPUSL achieved excellent UV-blocking performance, capable of shielding nearly all UV radiation while maintaining relatively high transparency (Fig. 6C and D). Figure 6E further confirms the 100% UV-blocking efficacy along with infrared-blocking capability. Moreover, COPUSL outperforms UV-blocking rates reported in the literature (Fig. 6F). This excellent performance is attributed to the aromatic structure of lignin and the presence of numerous phenolic groups, ketone groups, and intramolecular hydrogen bond (Fig. 6G). COPUSL1 was subjected to a 168-h accelerated UV aging experiment (Fig. S29). The results show that the color changed from its original light yellow to a brownish yellow, which is attributed to the susceptibility of urethane bonds to molecular chain scission during accelerated UV aging, followed by oxidation-induced yellowing. The whiteness of the original COPUSL1 was −20.6, and it was −21.1 after aging for 168 h, with little change in whiteness. The aged COPUSL1 remained smooth and flat without cracking, indicating good long-term durability.

## Conclusion

In summary, this work proposes a supramolecular polyurethane structural design strategy that utilizes the natural structural characteristics of lignin and castor oil. The resulting polyurethane comprises a “dynamic switch” constructed from dynamic disulfide bonds and hydrogen bonds, together with a “rigid–flexible balanced network” composed of castor oil long fatty chains and polyphenol lignin. This architecture synergistically combines tunable mechanical performance, rapid dynamic responsiveness at the molecular level, and multifunctional performance. The regulatory roles of the “dynamic switch” and “rigid–flexible balanced network” on COPUSL were systematically investigated, elucidating their synergistic effects in governing mechanical performance, thermodynamic behavior, self-healing characteristics, and energy dissipation. Furthermore, by ingeniously leveraging the efficient photon-energy-harvesting performance and rapid thermal conductivity derived from the ordered stacking of lignin molecules, the developed material system demonstrates high-efficiency UV-blocking performance and exceptional photothermal–electrical conversion. This work opens new pathways for the application of bio-based polyurethane in advanced manufacturing and sustainable environmentally responsive fields, offering valuable insights into the high-value utilization of bio-based feedstocks and the design of next-generation multifunctional smart materials.

## Materials and Methods

Information about the materials and methods used in this research is available in the Supplementary Materials.

## Data Availability

Additional data are available from the corresponding authors upon request. Data supporting the findings of this study are available within the article and its Supplementary Materials.

## Supplementary Materials

Supplementary 1 Experimental Section Schemes S1 and S2 Figs. S1 to S29 Tables S1 to S5
