# Supplementary material for: Tailoring Supramolecular Polyurethane Featuring Bio-based Rigid–Flexible Segment Hybrids with Intrinsic Photothermal Conversion and Ultraviolet Blocking
Source: Research (Wash D C). 2026 May 12;9:1282. doi: 10.34133/research.1282 (PMC13161532; doi:10.34133/research.1282)
Supplement: Supplementary 1 — Experimental Section Schemes S1 and S2 Figs. S1 to S29 Tables S1 to S5 [file research.1282.f1.docx]

You Title

Tailoring [Supramolecular](https://onlinelibrary.wiley.com/doi/abs/10.1002/smll.202307186) Polyurethane Featuring Bio-based Rigid-Flexible Segment Hybrids with Intrinsic Photothermal Conversion and UV-Blocking

Authors

Yun Hu^1^, Ye Sha^2^, Yan Fang^1, 3^, Xingxiang Liu^3^, Meng Zhang^1^, Puyou Jia^1*^, Guodong Feng, Liang Yuan^4*^, Lin Dai^5*^, Yonghong Zhou^1*^

Affiliations

^1^Institute of Chemical Industry of Forest Products, Chinese Academy of Forestry, 16 Suojin North Road, Nanjing 210042, China. E-mail: [jiapuyou@icifp.cn](mailto:shaye@njfu.edu.cn), [zyh@icifp.cn](mailto:zyh@icifp.cn).

^2^Department of Chemistry and Material Science, College of Science, Nanjing Forestry University, Nanjing 210037, China

^3^College of Chemical Engineering, Nanjing Forestry University, Nanjing 210037, China

^4^Anhui Provincial Engineering Center for High Performance Biobased Nylons, Biomass Molecular Engineering Center, School of Materials and Chemistry, Anhui Agricultural University, Hefei, Anhui, 230036, China. E-mail: yuanliang2020@ahau.edu.cn

^5^Tianjin Key Laboratory of Pulp and Paper, Tianjin University of Science and Technology, Tianjin 300457, China. E-mail: dailin@tust.edu.cn

**[1. Experimental Section 2](#_Toc29264)**

**[1.1 Materials 2](#_Toc19819)**

**[1.2 Preparation of phenol lignin 2](#_Toc15317)**

**[1.3 Synthesis of lignin-based polyurethane 3](#_Toc4881)**

**[1.4 Testing and characterization 4](#_Toc223)**

**[2. Results and discussion 7](#_Toc3103)**

**Fig.S1** ¹H NMR spectra of lignin, TALD-lignin, and phenol lignin.

**Fig.S2** HSQC NMR of lignin, TALD-lignin, and phenol-lignin.

**Fig.S3** FT-IR spectra of lignin, TALD-lignin, and phenol-lignin.

**Fig.S4** (a) ^31^P NMR spectra of TALD-lignin and phenol-lignin. (b) Yields of phenolation for different lignins in acid conditions.

**Fig. S5** FT-IR spectra of COPUSLs.

**Fig. S6** DTG of the COPUSLs.

**Fig. S7** XRD curves of the different COPUSL polyurethane.

**Fig. S8** Storage modulus of COPUSLs.

**Fig. S9** (a-d) The surface morphology of the COPUSL(1-4).

**Fig. S10** Tensile strength and elongation at break of COPUSLs.

**Fig. S11** Comparison of tensile strength and elongation at break of COPUSL with those of previously reported polyurethanes.

**Fig. S12** Cyclic tensile curve of COPUSLs.

**Fig. S13** Frequency sweep results of COPUSL1-2.

**Fig. S14** Temperature dependence of the storage modulus G’ and loss modulus G’’ of the COPUSLs.

**Fig. S15** FT-IR spectra of the C═O stretching vibration region in COPUSLs.

**Fig. S16** Variable-temperature FT-IR spectra of COPUSL2-4 heated from 25°C to 165°C in the ranges of 1850–1600 cm⁻¹ and 3600–3000 cm⁻¹.

**Fig. S17** 2D FTIR results of the synchronous spectr and the asynchronous spectra of (a-b) COPUSL2, (c-d) COPUSL3, (e-f) COPUSL4.

**Fig. S18** Molecular dynamics simulation visualization of COPUSL3 and COPUSL4. **Fig. S19** Stress-strain curves of COPUSL3 after multiple recycling processes.

**Fig. S20** Images of COPUSL1 before (a) and after self-healing (b-d) under heating at 120 °C for 5, 10, and 20 min, respectively.

**Fig. S21** Images of COPUSL3-4 before and after self-healing under heating at 120°C for 10 minutes.

**Fig. S22** Shape memory images of COPUSL3 recorded via infrared thermal imaging during xenon lamp irradiation.

**Fig. S23** Shape memory curves of COPUSL1.

**Fig. S24** Shape memory curves of COPUSL2.

**Fig. S25** Shape memory curves of COPUSL3.

**Fig. S26** Shape memory curves of COPUSL4.

**Fig. S27** 7-day solvent resistance of COPUSL3 (toluene, water, ethyl acetate, ethanol, petroleum ether, sulfuric acid, 1 mol/L NaOH, and 1 mol/L HCl).

**Fig. S28** Comparison of photothermal Conversion efficiency with typical additive-type polymers^[8-11]^.

**Fig. S29** The surface morphology of COPUSL1 before and after UV aging for 168 h (3 parallel experiments).

**Table S1** Composition of COPUSL.

**Table S2** Transparency, gel contents and DMA of the COPUSL.

**Table S3.** Mechanical properties of COPUSL.

**Table S4** Detailed information on the ATR-FTIR spectra of COPUSL.

**Table S5** Shape memory properties of the COPUSL.

1. **Experimental Section**

**1.1 Materials**

Xylose-extracted corncob residue was provided by Jinan Shengquan Group Co., Ltd. (Jinan, China). Terephthalaldehyde (TALD, 98%), isophorone diisocyanate (IPDI, 99%), dibutyltin dilaurate (DBTDL, 95%), bis(4-aminophenyl) disulfide (AD, 98%), and sodium bicarbonate (NaHCO₃, ≥99.5%) were purchased from Macklin Biochemical Co., Ltd. (Shanghai, China). Phenol (Ph-OH, 98%), hydrochloric acid (37% w/w, HCl), and sulfuric acid (98% w/w, H₂SO₄) were produced by Tianjin Jingdong Tianzheng Precision Chemical Reagent Factory. Deuterated chloroform (99.8%, CDCl₃), dimethyl sulfoxide-d6 (DMSO-d6), and 2-chloro-4,4,5,5-tetramethyl-1,3,2-dioxaphospholane (TMDP) were purchased from Sigma-Aldrich. Castor oil (CO) and diazoalkyl urea (DU, 98%) were obtained from Shanghai Aladdin Biochemical Technology Co., Ltd. N,N-Dimethylformamide (DMF, AR) was purchased from Nanjing Chemical Reagent Co., Ltd. All commercial chemicals were of analytical grade and used without further purification.

**1.2 Preparation of phenol lignin**

Phenol lignin was prepared from xylose-extracted corncob residue using the terephthalaldehyde protection method. A mixture of 4.8 g of xylose-extracted corncob residue, 8.0 g of terephthalaldehyde, and 25.0 mL of 1,4-dioxane was stirred until the aldehyde was completely dissolved. After complete dissolution of the aldehyde, 0.8 mL of hydrochloric acid was added, and the temperature was raised to 85 °C. The reaction was then carried out under a nitrogen atmosphere for 3 h, cooled to room temperature, and 1.15 g of NaHCO₃ was added with stirring for 45 min. After the reaction, the mixture was filtered, and the solid residue was washed with 1,4-dioxane. The filtrate was evaporated using a rotary evaporator. The solid obtained after rotary evaporation was redissolved in fresh 1,4-dioxane and precipitated with 400 mL of diethyl ether under stirring for 1 h, allowing most carbohydrates and unreacted terephthalaldehyde to dissolve. The product was then filtered and purified using a Soxhlet extractor with diethyl ether as the solvent to remove all impurities. The solid was dried overnight in a vacuum oven at 45 °C to obtain terephthalaldehyde-lignin (TALD-Lignin).

Next, 3.0 mL of phenol was pre-melted, and 0.5 g of TALD-Lignin was added. Then, 0.108 mL of concentrated H₂SO₄ was added dropwise, and the reaction was conducted at 110 °C for 30 min. After cooling to room temperature, 5 mL of DMSO was added. The resulting solution was dropwise added to 300 mL of deionized water, which had been previously acidified to pH = 1 using H₂SO₄. After precipitation, the phenolized lignin was filtered, washed with water until the filtrate reached a neutral pH, and then the collected solid was dried overnight in a vacuum oven at 45°C to obtain phenol lignin (M_n_ = 1096 g/mol).


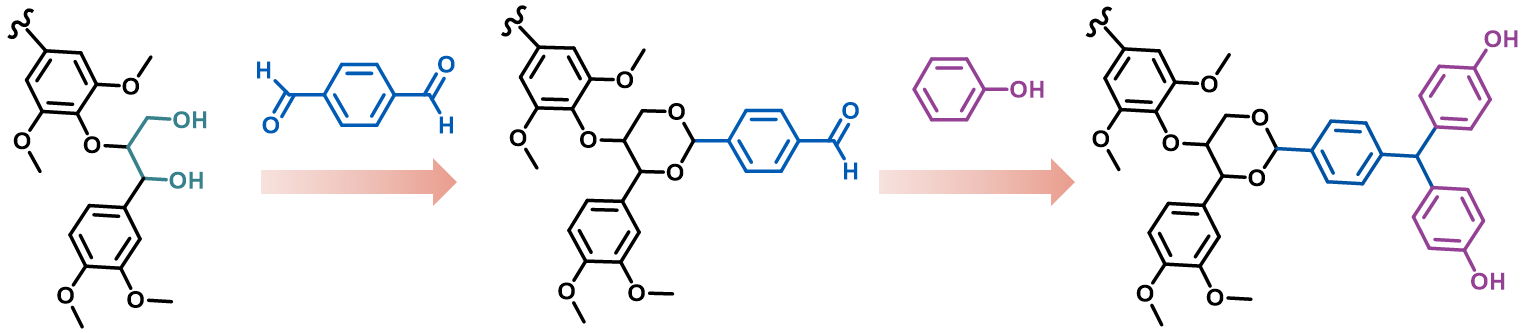


**Scheme S1** Synthesis reaction of phenol lignin

**1.3 Synthesis of lignin-based polyurethane**

Castor oil (10.26 g, 11 mmol) and the solvent DMF were added to a four-necked flask. The mixture was heated to 60 °C, after which IPDI (4.45 g, 20 mmol) and one drop of the catalyst DBTDL were added. The reaction was carried out under a nitrogen atmosphere for 60 min to obtain the intermediate CO-IPDI. The chain extender AD (0.875 g, 3.65 mmol) was then added, and the reaction continued for 2 h to yield the sulfur-containing castor oil-based polyurethane prepolymer CO-IPDI-AD. Due to the rigid structure and multiple hydroxyl groups of phenol-lignin, which provide additional cross-linking sites for the formation of a dynamic covalent network, phenol-lignin (0.18 mmol) was added, and the reaction proceeded for another 2 h, ultimately yielding the lignin-based polyurethane COPUSL. By varying the amounts of -NH₂ (from AD) and -OH (from phenol ligninL), a series of lignin-modified polyurethane materials were prepared and labeled as COPUSLX. The polymer was poured into a polypropylene mold, left at room temperature for 12 h, and then dried in an oven at 70–80 °C for 12 h to obtain the cured COPUSL resins. The formulations of the LPCOPU materials are listed in Table S1.

**1.4 Testing and characterization**

FT-IR spectra of the samples were captured using a Nicolet iS10 Fourier Transform Infrared Spectrometer manufactured by Thermo Fisher Scientific, USA. ¹H NMR testing was performed using a Bruker AVANCE3 (400 MHz) spectrometer with deuterated chloroform as the solvent. The gel content (C_gel_) was determined using the Soxhlet extraction method (ASTM D2765). A sample mass (W₀, 1.5 g) was placed in 25 mL of acetone and heated at 60 °C for 24 h. It was then dried at 80 °C and reweighed to obtain W₁. The gel content was calculated using the following formula:

C_gel_ = (W₁ / W₀) × 100%

where W₀ and W₁ represent the initial and reweighed masses, respectively.

Differential Scanning Calorimetry (DSC) testing was conducted on a DSC8000 thermal analyzer from PerkinElmer Corporation, USA. The samples were subjected to two scanning cycles under a N₂ atmosphere at a heating rate of 20 °C per minute over a temperature range of -50 to 100 °C. Thermogravimetric Analysis (TGA) was performed on an STA 409PC thermogravimetric analyzer (Netzsch, Germany) from 25 °C to 800 °C at a heating rate of 20 °C per minute. Contact angles were measured using a JC2000C1 instrument (Powereach, China). The light transmittance of the films was analyzed by UV-Vis spectrophotometry, focusing on wavelengths from 400 to 800 nanometers. The surface morphology of the obtained material was examined using a Hitachi-3400 Type I scanning electron microscope (SEM). The UV aging test was conducted in a box-type UV accelerated aging tester, model HYW-UV340 (Hongjin Instrument, Guangdong Hongyu Group). The UV irradiation time was 168 h, the temperature was 70 °C, and the irradiance was 0.48 W/m². Photos of the aged samples were taken every 24 h. Tensile testing was performed using a UTM 4304 (Suns, China) (ASTM D638). For the uniaxial tensile and loading-unloading cycle tests of rectangular specimens, the strain rate was set to 50 mm/min. For accuracy, five tests were conducted for each sample. Cyclic tensile tests involved continuous loading-unloading tests without rest intervals within a maximum strain range of 50% to 300% to evaluate the energy dissipation capacity of the samples. The toughness (τ) of the elastomer samples is defined by integrating the area under engineering stress-strain curves (σ-ε), measured at the stretching speed of 100 mm·min-1, according to the following equation:

$$\tau=\int_{\varepsilon=0}^{\varepsilon=\varepsilon_{\max}} \sigma d\varepsilon$$

where σ and ε are the engineering stress and engineering strain respectively, and εmax is the elongation at the break of the elastomer samples.

The samples were cut into small rectangular strips and immersed in different solvents for one week, with their condition observed over time. Shape memory testing was conducted using a Philips FLIR thermal imaging camera, while the thermal behavior was recorded with an iPhone camera. Optical microscopy observations were performed using a Leica ICC50W optical microscope (Germany). A cross-shaped incision was made on the sample surface, and the sample was then subjected to a recovery process at 120 °C. The self-healing efficiency was calculated as follows:

η = (L_original – L_heal) / L_original × 100%

Here, η represents the self-healing rate, L_original is the original length of the incision, and L_heal is the length after the healing process. The COPUSL samples were crushed and placed in a rectangular mold. They were then hot-pressed at 150 °C under 0.2 MPa pressure for 40 min to obtain the recycled polyurethane samples. The mechanical properties of the recycled samples were measured, with each group tested no fewer than three times.

UV-vis transmittance spectra were recorded on a UV-2550 Spectrophotometer (Shimadzu) (ISO 13468-1). XRD were conducted on an XRD-D8 FOCUS (Bruker, Germany) with Cu Kα radiation, with data ranging from 10° to 70° and scanning speed of 10 °·min^-1^.

DMA was carried out on the DMA Q800 (TA Instrument, USA) under the tension mode (temperature range of -100 to 100 °C and a heating rate of 10 °C·min^-1^). Rectangular elastomer samples (length, width, thickness: 20.0 × 4.0× 0.4 mm) were tested at a strain of 0.1% and a frequency of 1 Hz. TG was performed to realize the thermal stability of the polyurethane on a TA Q500 instrument. The temperature was heated from room temperature to 800 °C at a heating rate of 10 °C·min^-1^ under a N_2_ atmosphere. Rheology experiments were performed on Micro infrared combined rheometer (MAS60) using the disk-type specimen with a size of about 20 ± 1 mm in diameter and 1 ± 0.05 mm in thickness. The temperature sweep experiments were conducted with a temperature range of 25 to 125 °C with a strain of 1%, a normal force of 1 N, and a frequency of 1 Hz. The frequency sweep experiments were performed over a frequency range of 0.1 to 100 rad·s^-1^ at temperatures from 25 to 150 °C. The main curves of G’ and G’’ were obtained using the time temperature equivalence principle with 50 °C as the reference temperature.

The absorption data was obtained via UV-3600i Plus UV-vis-NIR spectrophotometer (Shimadzu, Japan) with the wavelength range from 200–2500 nm.

A xenon light (Ciamite BBZM-Ⅰ, China) and infrared laser (Xiai MDL-III-808/1~2500mW, China) were used as the light source. Temperature changes and the temperature data were collected every 1 s. The photothermal conversion performance was determined according to previous method1.

The molecular-level packing behavior and intermolecular interactions were investigated through molecular dynamics (MD) simulations and energy decomposition analysis. All calculations were performed using the Forcite module. The condensed-phase optimized molecular potentials for atomistic simulation studies (COMPASS III) forcefield was applied for all calculations. Atomic charges were assigned using the QEq method. Initially, an amorphous cell containing 10 chains was constructed. The system was first optimized using the Smart Minimizer algorithm to reach a stable conformation with low energy. Subsequently, an Anneal procedure was carried out to sample the conformational space and locate the global energy minimum. The equilibrated structure was then subjected to a series of MD simulations in the NVT, NPT, and NVE ensembles sequentially to further relax the system and ensure stability. The Nosé-Hoover thermostat was employed for temperature control during MD runs. The binding energies between several compounds were studied based on density functional theory (DFT). All calculations were carried on ORCA (version5.0.3) program and configuration optimization calculations were progress in the B3LYP-D3 and def2-SVP basis set levels, while def2-TZVP basis set with higher precision was used for single point energy calculation.[2] The binding energy Ebind was calculated as follows:

$$E_{\mathrm{Bind}}= E_{\mathrm{AB}}- E_{A}- E_{B}$$

EAB, EA, EB represent the energy of complex AB, pure A, and pure B, respectively.

1. **Results and discussion**


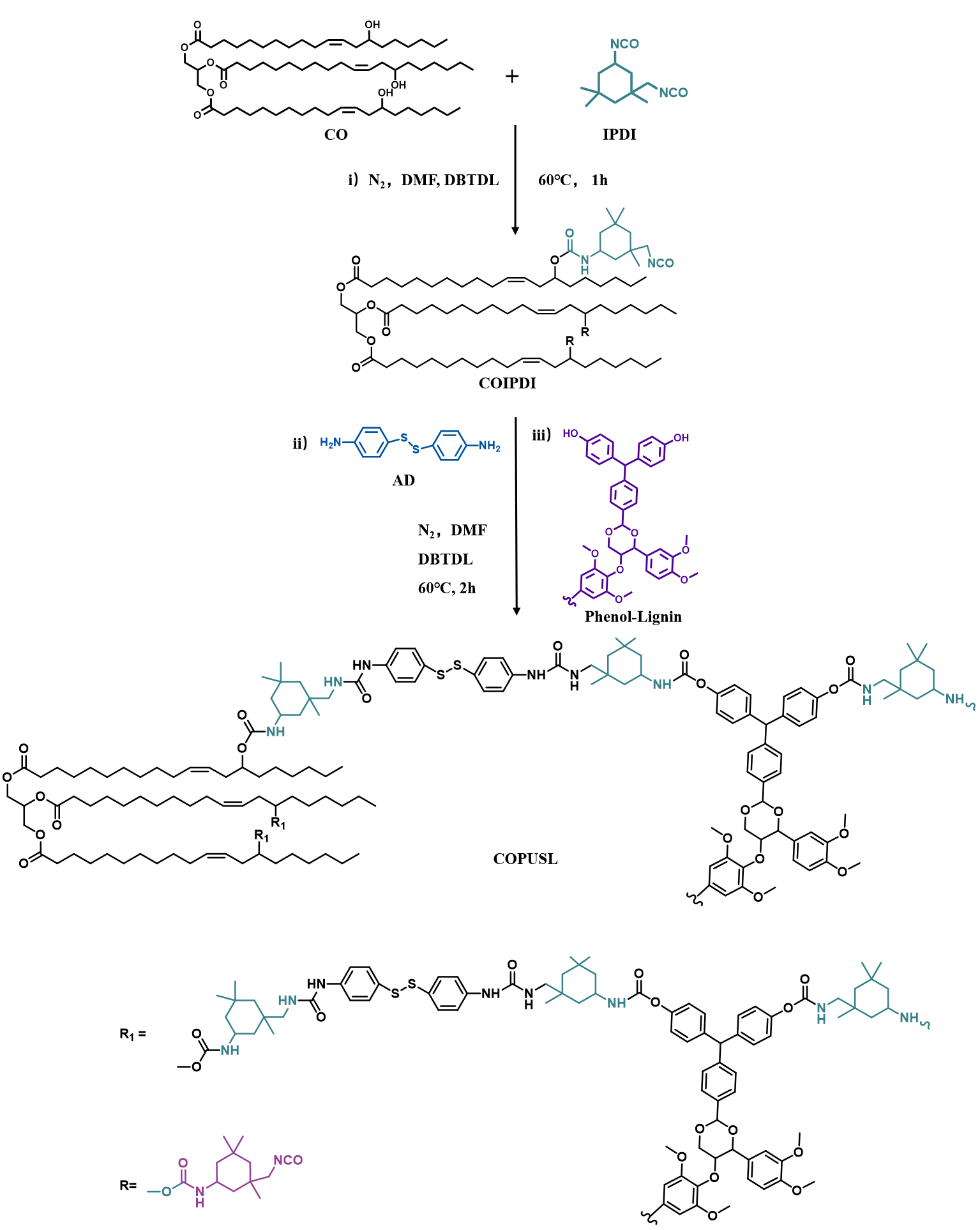


**Scheme S2** Synthesis route of COPUSLs

**Fig.S1** ¹H NMR spectra of lignin, TALD-lignin, and phenol lignin.


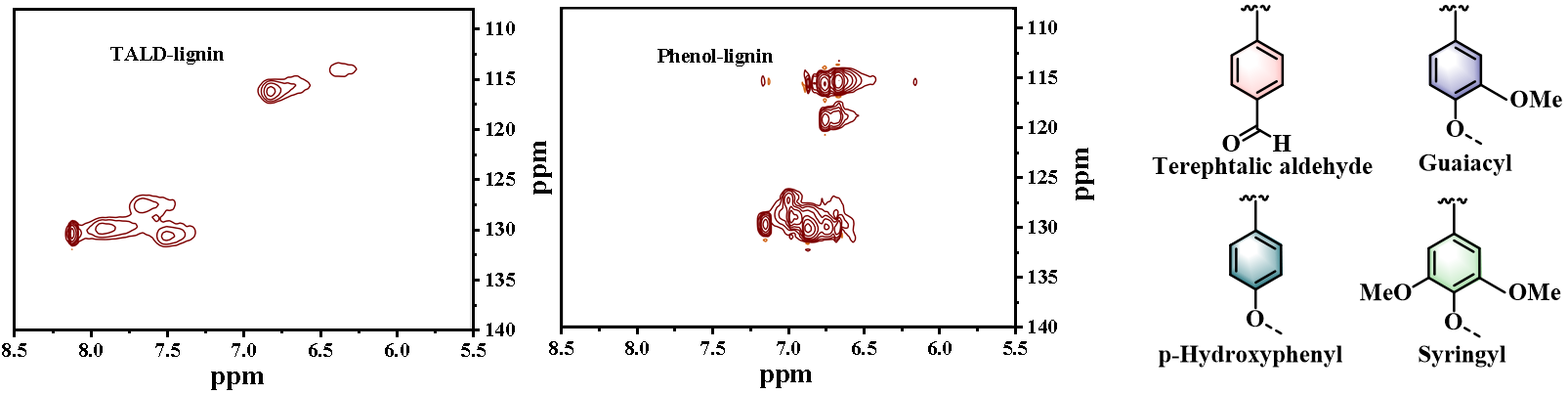


**Fig.S2** HSQC NMR of lignin, TALD-lignin, and phenol-lignin.

**Fig.S3** FT-IR spectra of lignin, TALD-lignin, and phenol-lignin.


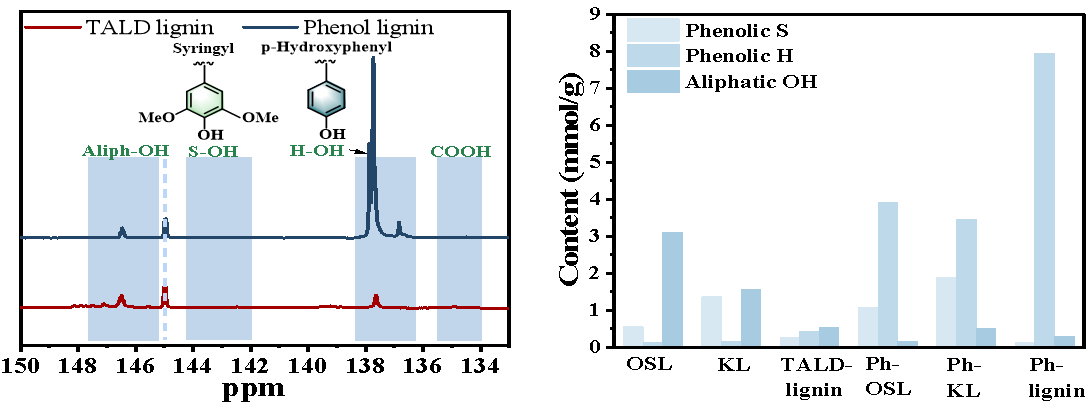


**Fig. S4** (a) ^31^P NMR spectra of TALD-lignin and phenol-lignin. (b) Yields of phenolation for different lignins in acid conditions.

**Fig. S5** FT-IR spectra of COPUSLs.

**Fig. S6** DTG of the COPUSLs.

**Fig. S7** XRD curves of the different COPUSL polyurethane.

**Fig. S8** Storage modulus of COPUSLs.


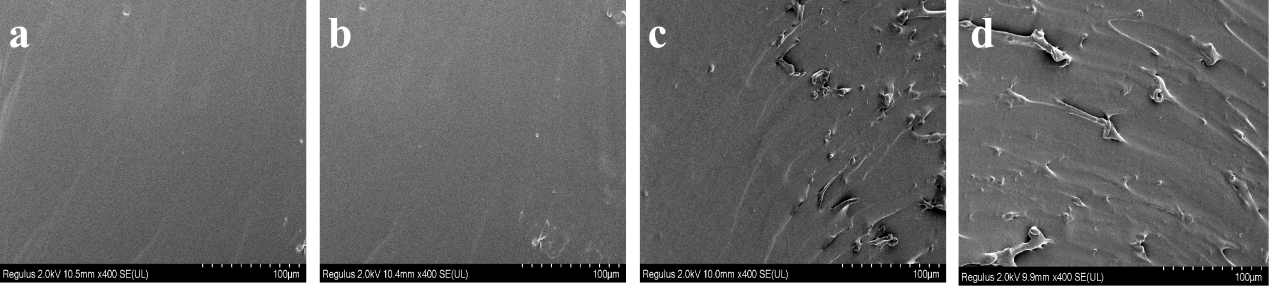


**Fig. S9 (a-d)** The surface morphology of the COPUSL(1-4) .

**Fig. S10** Tensile strength and elongation at break of COPUSLX.

**Fig. S11** Comparison of tensile strength and elongation at break of COPUSL with those of previously reported polyurethanes^[1-7]^.


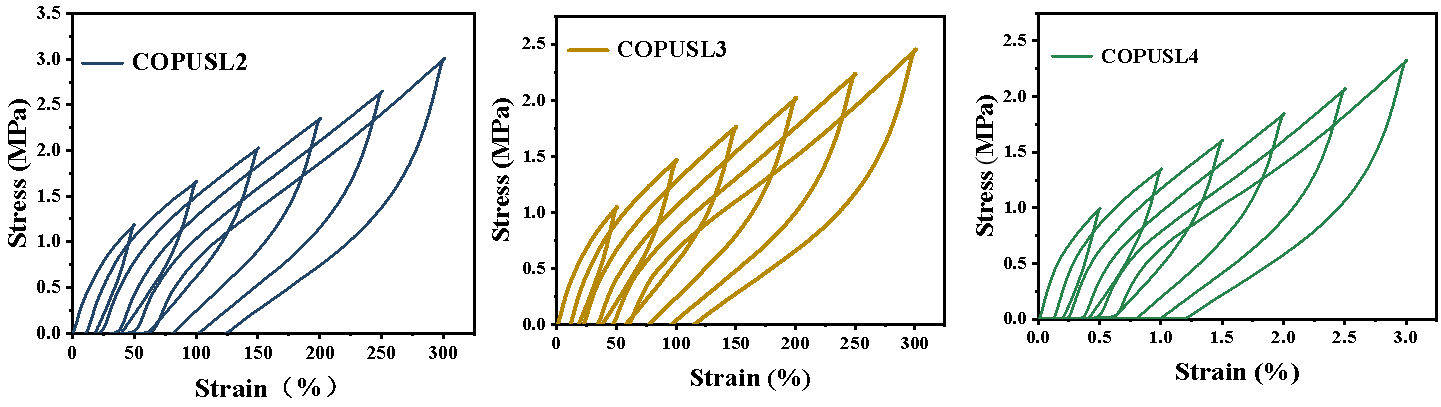


**Fig. S12** Cyclic tensile curve of COPUSLs.


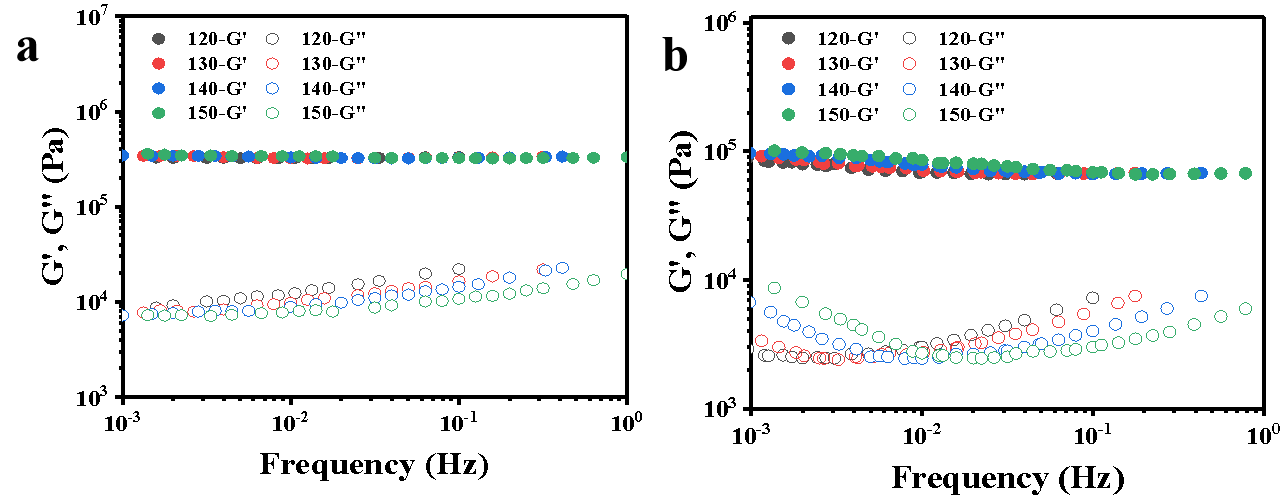


**Fig. S13** Frequency sweep results of COPUSL1-2.

**Fig. S14** Temperature dependence of the storage modulus G’ and loss modulus G’’ of the COPUSLs.


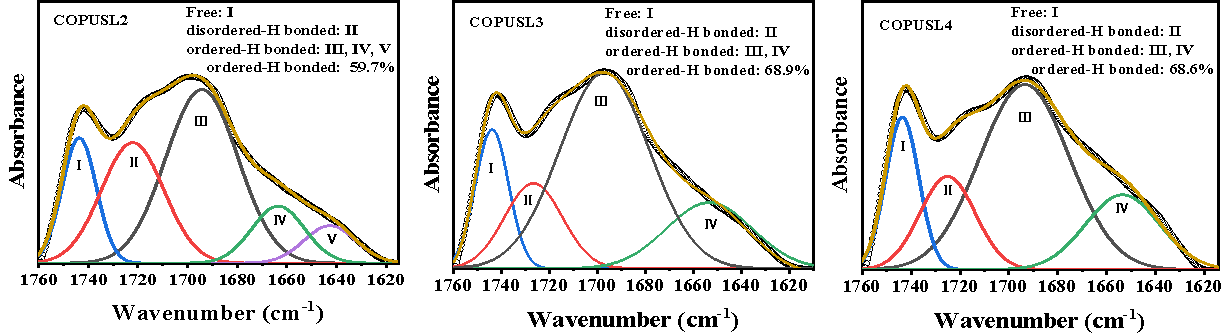


**Fig. S15** FT-IR spectra of the C═O stretching vibration region in COPUSLs.


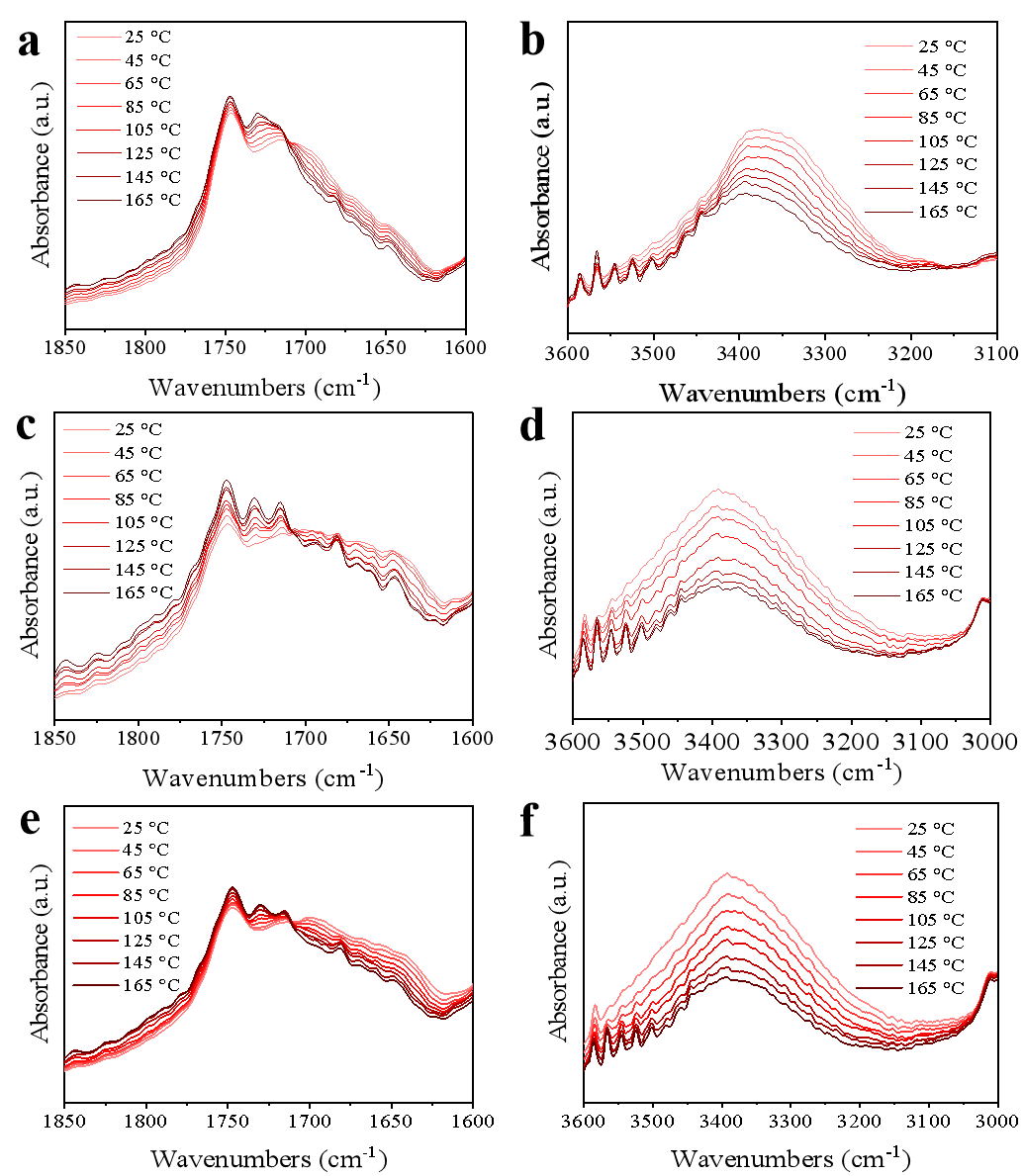


**Fig. S16** Variable-temperature FT-IR spectra of COPUSL2 (a-b), COPUSL3 (c-d), COPUSL4 (e-f) heated from 25°C to 165°C in the ranges of 1850–1600 cm⁻¹ and 3600–3000 cm⁻¹.


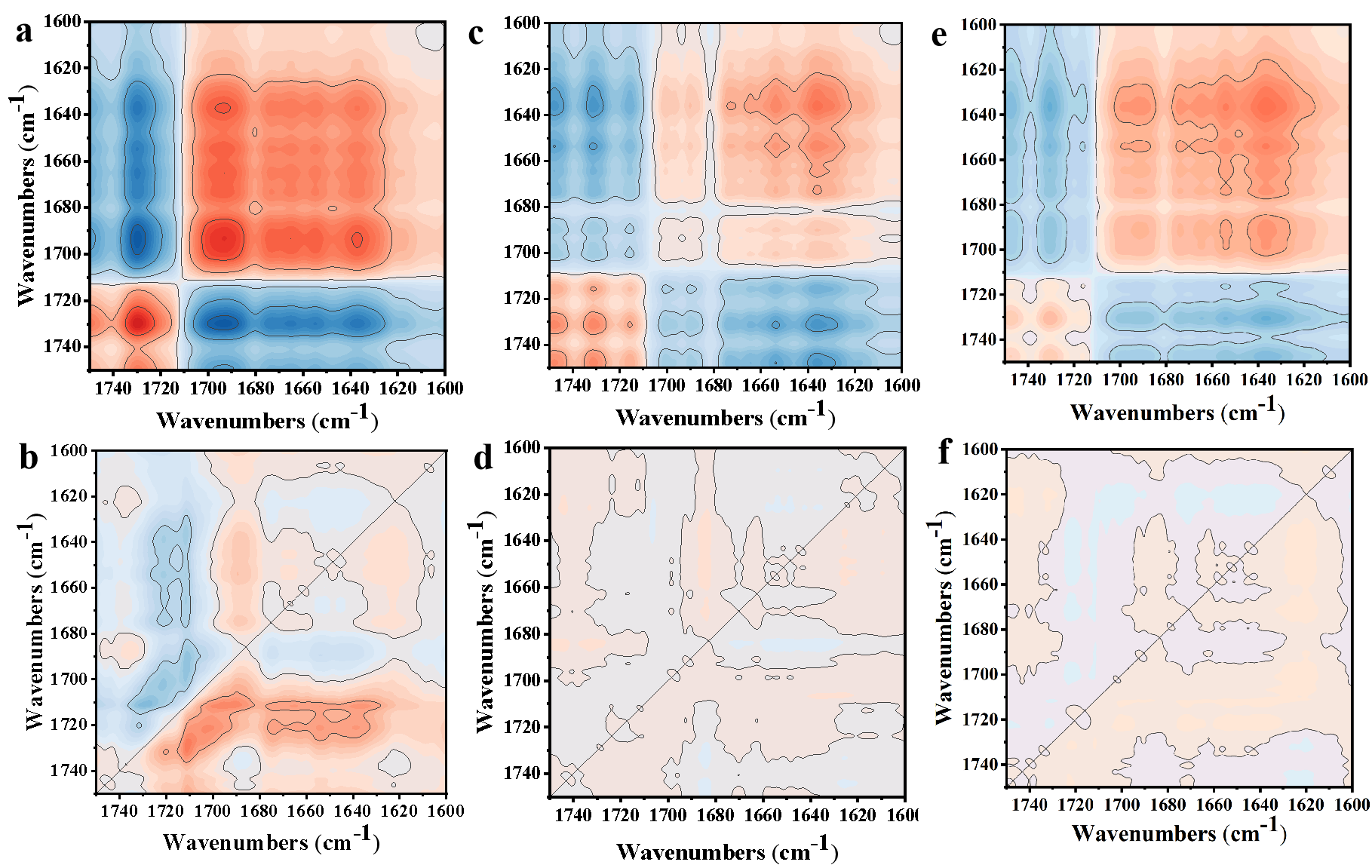


**Fig. S17** 2D FTIR results of the synchronous spectr and the asynchronous spectra of (a-b) COPUSL2, (c-d) COPUSL3, (e-f) COPUSL4.


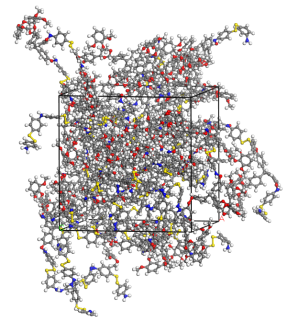

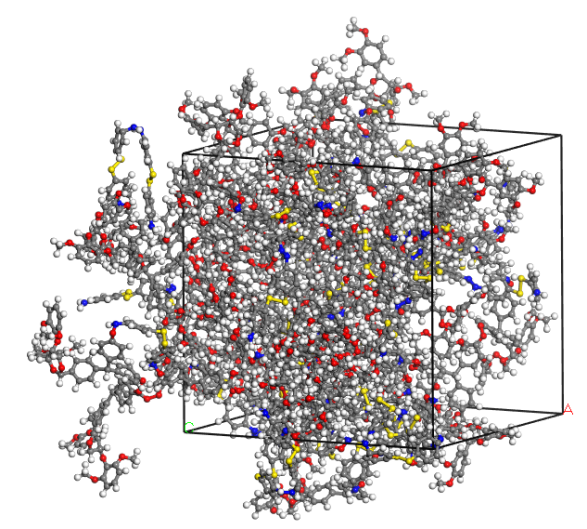


**Fig. S18** Molecular dynamics simulation visualization of COPUSL3 and COPUSL4.

**Fig. S19** Stress-strain curves of COPUSL3 after multiple recycling processes.


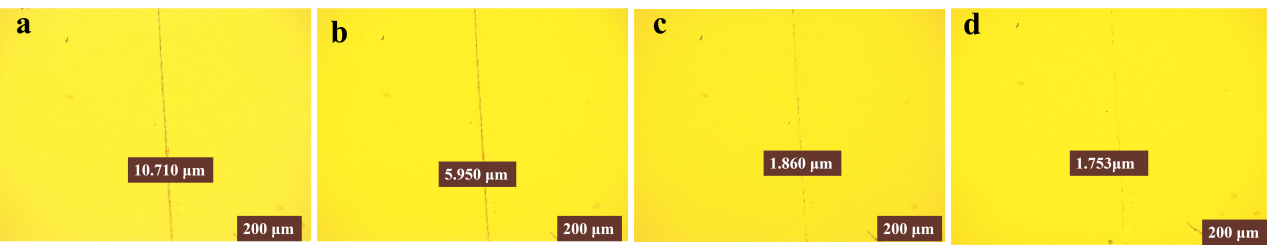


**Fig. S20** Images of COPUSL1 before (a) and after self-healing (b-d) under heating at 120 °C for 5, 10, and 20 min, respectively. The results showed that with heating times of 5, 10, and 20 min, the self-healing rate was 44.44%, 82.63%, and 83.63%, respectively. Considering time and cost, 10 min is the optimal self-healing time.


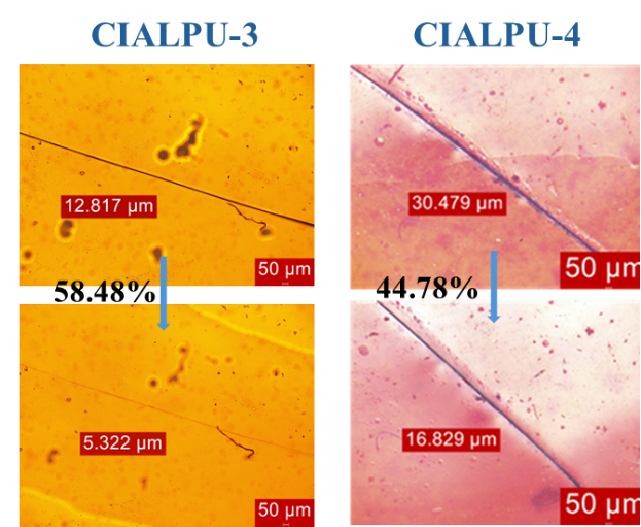


**Fig. S21** Images of COPUSL3-4 before and after self-healing under heating at 120°C for 10 minutes.


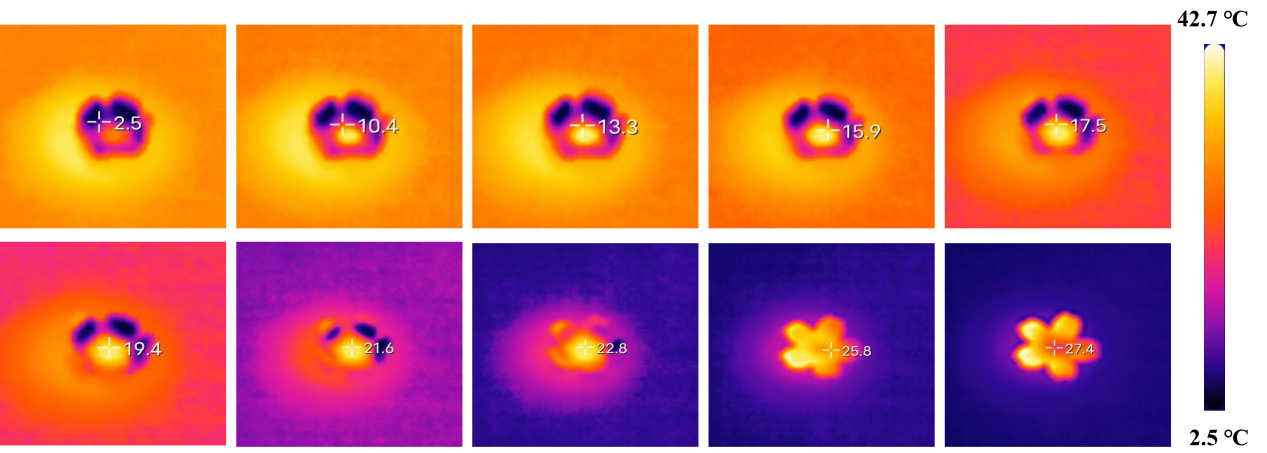


**Fig. S22** Shape memory images of COPUSL3 recorded via infrared thermal imaging during xenon lamp irradiation.


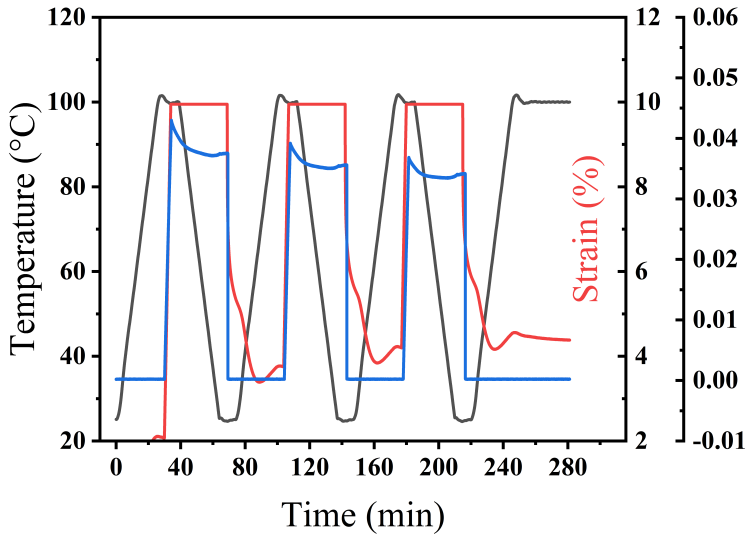


**Fig. S23** Shape memory curves of COPUSL1.


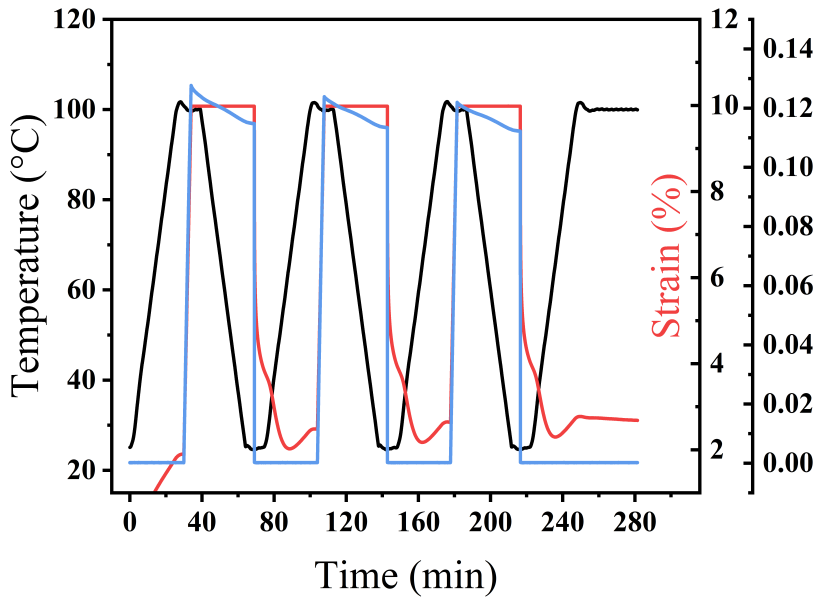


**Fig. S24** Shape memory curves of COPUSL2.


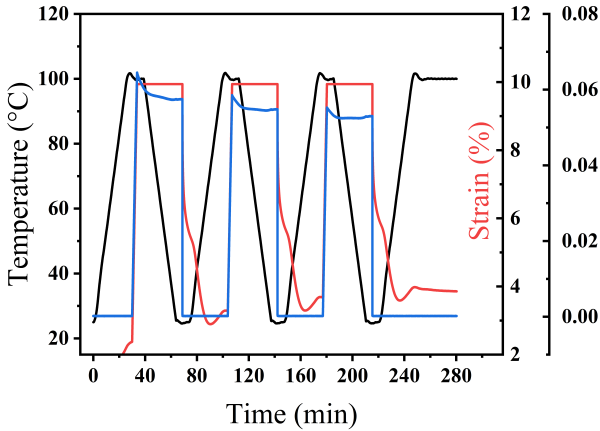


**Fig. S25** Shape memory curves of COPUSL3.


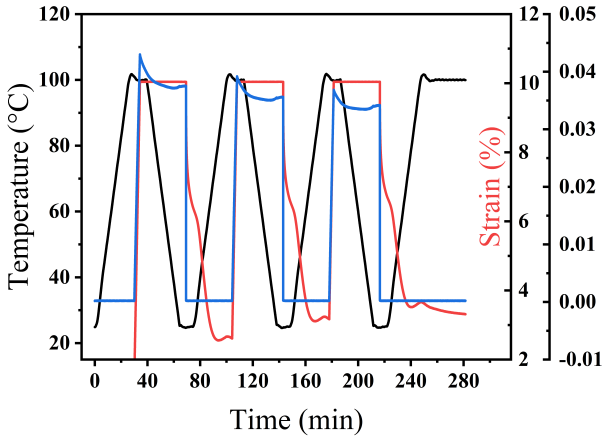


**Fig. S26** Shape memory curves of COPUSL4.


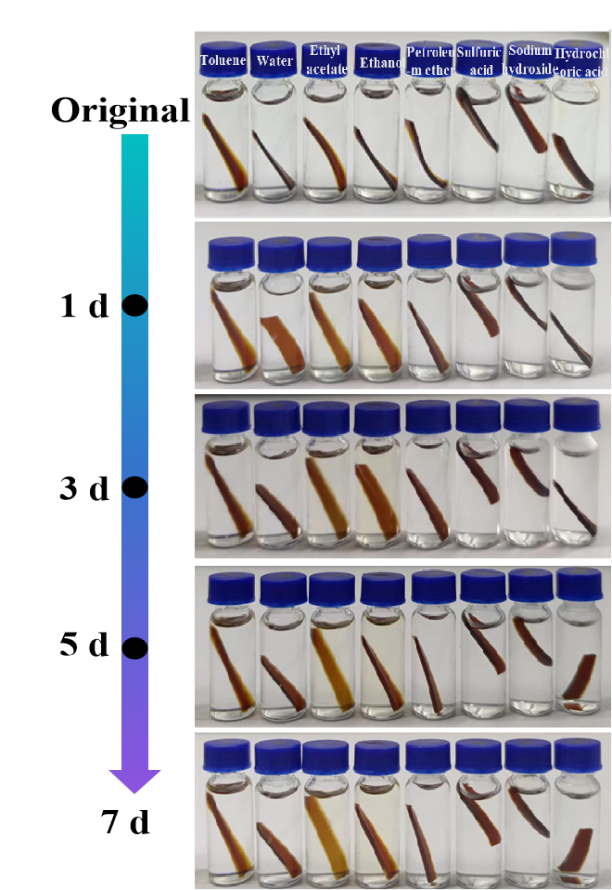


**Fig. S27** 7-day solvent resistance of COPUSL3 (toluene, water, ethyl acetate, ethanol, petroleum ether, sulfuric acid, 1 mol/L NaOH, and 1 mol/L HCl).


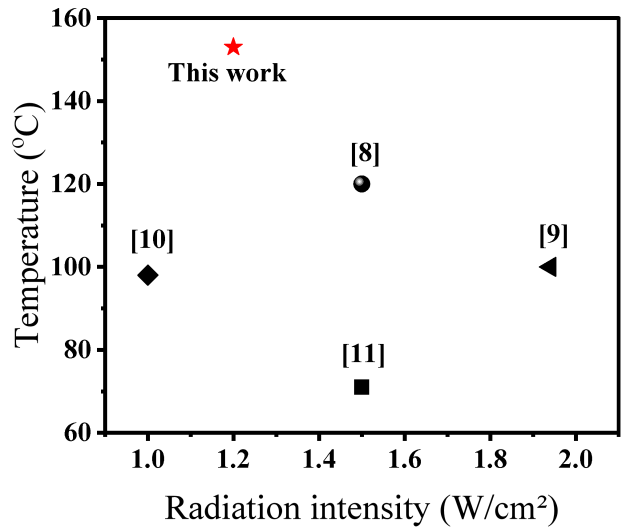


**Fig. S28** Comparison of photothermal conversion efficiency with typical additive-type polymer systems^[8-11]^.


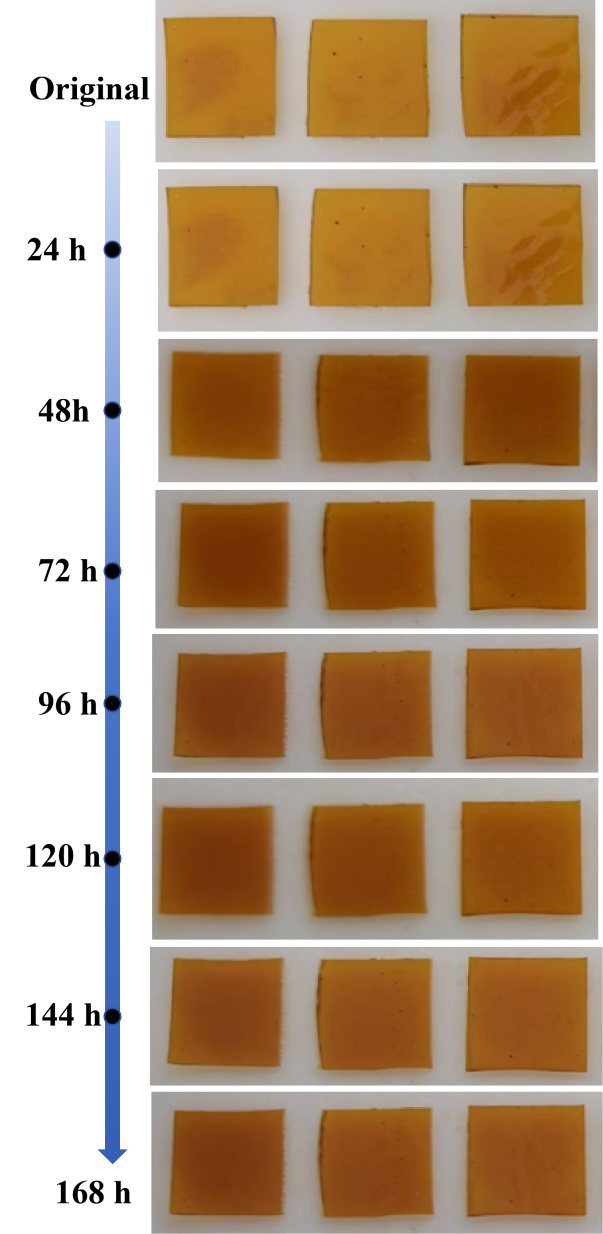


**Fig. S29** The surface morphology of COPUSL1 before and after UV aging for 168 h (3 parallel experiments).

**Table S1** Composition of COPUSL.

| **Samples** | **CO (mmol)** | **IPDI (mmol)** | **AD (mmol)** | **Phenol lignin (mmol)** |
| --- | --- | --- | --- | --- |
| COPUSL1 | 11 | 20 | 3.15 | 0.18 |
| COPUSL2 | 11 | 20 | 3.15 | 0.36 |
| COPUSL3 | 11 | 20 | 3.15 | 0.55 |
| COPUSL4 | 11 | 20 | 3.15 | 0.72 |

**Table S2** Transparency, gel contents and DMA of the COPUSL.

| **Samples** | **Gel contents (%)** | ***E_25_^a^***  **(MPa)** | ***E_Tg+50_^b^***  **(MPa)** | ***v_e_***  **(mol/m^3^)** |
| --- | --- | --- | --- | --- |
| COPUSL1 | 98.07 ± 0.37 | 18.8 | 2.82 | 351.25 |
| COPUSL2 | 99.47 ± 0.20 | 41.1 | 3.68 | 453.60 |
| COPUSL3 | 97.70 ± 0.47 | 81.3 | 9.87 | 1220.41 |
| COPUSL4 | 96.74 ± 0.14 | 21.6 | 2.60 | 319.48 |

*^a^* Storage modulus of 25 °C. *^b^* Storage modulus of T_g_ + 50 °C.

**Table S3.** Mechanical properties of COPUSL.

| **Samples** | **Tensile strength /MPa** | **Young’s modulus /MPa** | **Elongation**  **at break /%** | **Toughness**  **/J·m^-3^** |
| --- | --- | --- | --- | --- |
| COPUSL1 | 5.4 ± 0.5 | 0.64 ± 0.0.2 | 675.48 ± 33 | 18.2 ± 1.3 |
| COPUSL2 | 9.3 ± 1.1 | 0.89 ± 0.04 | 701.4 ± 41 | 28.1 ± 1.6 |
| COPUSL3 | 8.8 ± 0.5 | 1.17 ± 0.1 | 635.1 ± 54 | 28.8 ± 1.5 |
| COPUSL4 | 7.1 ± 0.4 | 1.09 ± 0.1 | 557.1 ± 42 | 22.0 ± 0.9 |

**Table S4** Detailed information on the ATR-FTIR spectra of COPUSL.

| **Samples** | **Bond type** | **Urethane,**  **free** | **Urethane,**  **H-bond,**  **ordered** | **Urea,**  **free** | **Urea，**  **H-bond,**  **disordered** | **Urea，**  **H-bond,**  **ordered** |
| --- | --- | --- | --- | --- | --- | --- |
| COPUSL1 | Wavenumber (cm^-1^) | 1743 | 1719 | 1693 | 1665 | 1640 |
|  | Percentage (%) | 16.41 | 31.72 | 29.26 | 18.23 | 4.38 |
| COPUSL2 | Wavenumber (cm^-1^) | 1741 | 1713 | 1692 | 1670 | 1650 |
|  | Percentage (%) | 14.56 | 25.73 | 42.83 | 10.27 | 6.62 |
| COPUSL3 | Wavenumber (cm^-1^) | 1741 | 1713 | 1692 | 1670 | 1650 |
|  | Percentage (%) | 14.97 | 15.99 | 50.53 | 18.39 | - |
| COPUSL4 | Wavenumber (cm^-1^) | 1741 | 1713 | 1692 | 1670 | 1650 |
|  | Percentage (%) | 15.92 | 15.54 | 51.46 | 17.11 | - |

**Table S5** Shape memory properties of the COPUSL.

| **Samples** | | **COPUSL1** | **COPUSL2** | **COPUSL3** | **COPUSL4** |
| --- | --- | --- | --- | --- | --- |
| Cycle1 | R_f_ | 100% | 100% | 100% | 100% |
|  | R_r_ | 62% | 80% | 71% | 74% |
| Cycle2 | R_f_ | 100% | 100% | 100% | 100% |
|  | R_r_ | 58% | 79% | 67% | 69% |
| Cycle3 | R_f_ | 100% | 100% | 100% | 100% |
|  | R_r_ | 55% | 78% | 64% | 65% |

REFERENCES

1. Miao P, Liu J, He M, Leng X, Li Y. Bio-based non-isocyanate polyurethane with closed-loop recyclability and its potential application. Chem Eng J. 2023; 475: 146398.

2. Guo Y, Chen S, Sun L, Yang L, Zhang L, Lou J, You Z. Degradable and Fully Recyclable Dynamic Thermoset Elastomer for 3D‐Printed Wearable Electronics. Adv Funct Mater. 2020;31.

3. Vu V.-P, Kim S.-H, Mai V.-D, Ra S, An S, Lee S.-H. Bio-based conductive polyurethane composites derived from renewable castor oil with enhanced self-healing ability for flexible supercapacitors. J. Mater. Sci. Technol. 2024;188: 44-61.

4. Yang W, Qiu S, Zhang J, Cheng Z, Song L, Hu Y. Innovative design and green synthesis of bio-based non-isocyanate polyurethanes: Efficient combination of cardanol and carbon dioxide with high fire safety and robust adhesion. Chem Eng J. 2024;482: 148846.

5. Liu X, Yang X, Wang S, Wang S, Wang Z, Liu S, Xu X, Liu H, Song Z. Fully Bio-Based Polyhydroxyurethanes with a Dynamic Network from a Terpene Derivative and Cyclic Carbonate Functional Soybean Oil. ACS Sustain. Chem. Eng. 2021; 9: 4175-4184.

6. Xue Y, Zhang T, Peng H, Ma Z, Zhang M, Lynch M, Dinh T, Zhou Z, Zhou Y, Song P. Fire-retardant anti-dripping biodegradable and biobased polyurethane elastomers enabled by hydrogen-bonding with cellulose nanocrystals. Nano Research 2024; 17: 2186-2194.

7. Huang Z, Han D, Yi G, Lin W, Lin X, Sun Y, Wang H. High‐Performance and Multifunctional Lignin‐Derived Polyurethane Elastomers for Robotic Flexible Protective Layers. Adv Funct Mater.2025; 35.

8. Hou S, Wang J, Zhang G, Liu X, Wu Z, Liu B, Lu M, Zhang M, Qiu S, et al. An all-in-one Ag2Se-based flexible solar-thermoelectric generator with photothermal integration. *Nat commun.* 2026; 17.

9. Jiaojun T, Yingqi C, Wenlong X, Fangfang L, Meiyun Z, Shunxi S, Bin,Y, Jingyi N, Chunmei L. Aramid Nanofiber‐Based Multifunctional Phase Change Film for Photo/Electro‐Thermal Energy Conversion, Harvesting and Storage. *Small* 2026; 22.

10. Tang Y, Qian H, Xie C, Xia T, Wang C, Zhang C, Cai J, Bai X, Yu G, et al. Encapsulation of Carbon Nanotubes with Sulfonated Polymer for Photothermal Conversion of Fructose with Superior Productivity. *ACS Catalysis* 2026; 16, 2353-2362.

11. Xie W, Liang J, Chang G, Li R. 3D-architectured MXene/thermally expandable microsphere composites with synergistic thermal insulation and photothermal conversion. *Prog Org Coat.* 2026; 210, 109689.
